# *N,N*′-Di-Boc-2H-Isoindole-2-carboxamidine—First Guanidine-Substituted Isoindole

**DOI:** 10.3390/molecules27248954

**Published:** 2022-12-15

**Authors:** Petar Štrbac, Anamarija Briš, Davor Margetić

**Affiliations:** Laboratory for Physical-Organic Chemistry, Division of Organic Chemistry and Biochemistry, Ruđer Bošković Institute, 10001 Zagreb, Croatia

**Keywords:** cycloaddition, Diels–Alder reaction, heterocycles, organic synthesis, indoles, DFT calculations

## Abstract

Synthesis of *N,N*′-Di-Boc-2H-isoindole-2-carboxamidine, the first representative of isoindoles containing guanidine functionality, was carried out. The cycloaddition reactivity of this new Diels–Alder heterodiene was studied and the title compound was employed as a cycloaddition delivery reagent for guanidine functionality. Higher reactivity was found in comparison with the corresponding pyrrole derivative. Substitution with fluorine or guanidine functionality does not change the reactivities of isoindoles, and these findings are in good accord with computational results.

## 1. Introduction

**Figure 1:**
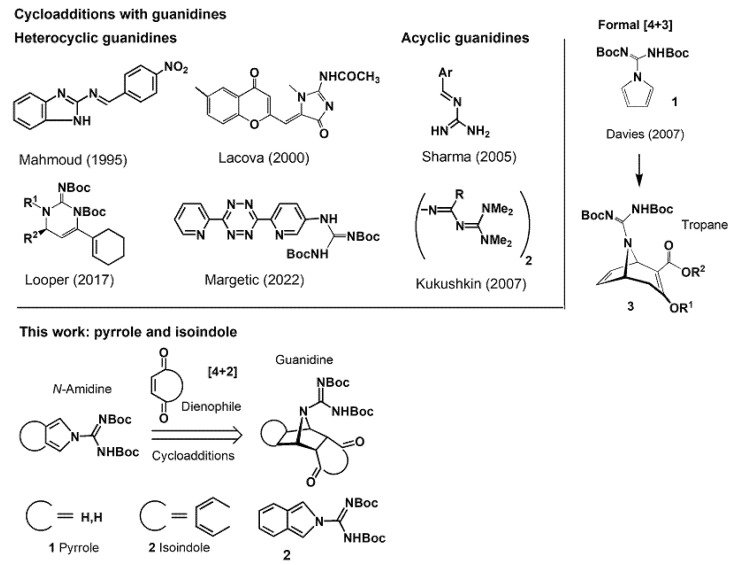
Used guanidines in Diels–Alder reactions and 2-carboxamidine dienes described in this work [6,7,13].

Guanidines are a class of nitrogen-containing molecules with very interesting physico-chemical properties, [1] especially very high basicity [2] and biological activity [3]. Hence, various aspects of guanidine chemistry were extensively studied computationally and experimentally, including their synthesis. The most viable synthetic routes towards polycyclic, complex organic molecules include the introduction of guanidine functionality at the later stages of multi-step synthesis, such as in tetrodotoxin synthesis [4,5]. One of the most efficient ways for the construction of polycyclic molecules is Diels–Alder (DA) cycloaddition; however, the cycloaddition approach to polycycles containing guanidine functionality has been rarely utilized. Diels–Alder cycloadditions, which involve diene or dienophile partners possessing guanidine functionality, were summarized in Figure 1 [6,7] which also depicts guanidine delivery cycloaddition reagents [8,9]. All of these are unsymmetrical diene molecules, and their cycloaddition reactions lead to the formation of unsymmetrical products.

For the purpose of our studies towards the synthesis of polycyclic molecules, symmetrical diene reagents were required, and, in view of earlier studies [10,11], pyrrole and isoindole *N*-carboxamidine derivatives **1** and **2** were selected. These molecules possess C_2v_ symmetry [12] and upon cycloaddition form guanidine functionality which is ‘protected’ in pyrrole moiety. Whereas pyrrole-2-carboxamidine **1** is a known compound, its DA cycloaddition properties were not reported. Only rhodium-catalyzed [4+3] cycloaddition was utilized to prepare tropane bicyclo [3.2.1] octane skeleton **3**, where pyrrole acted as a dipolarophile partner (Figure 1) [13]. Corresponding isoindole-2-carboxamidines have not been synthetized previously.

The objective of this work is to prepare isoindole-2-carboxamidine **2**, the first representative of an isoindole guanidine cycloaddition delivery reagent, and assess its cycloaddition properties experimentally and computationally. Our preliminary DFT computational study revealed that this approach is feasible and both pyrrole and isoindole dienes are predicted to have sufficient reactivity [14].

## 2. Results and Discussion

### 2.1. Cycloadditions of 1-Carboxamidine Pyrrole

**Scheme 1:**
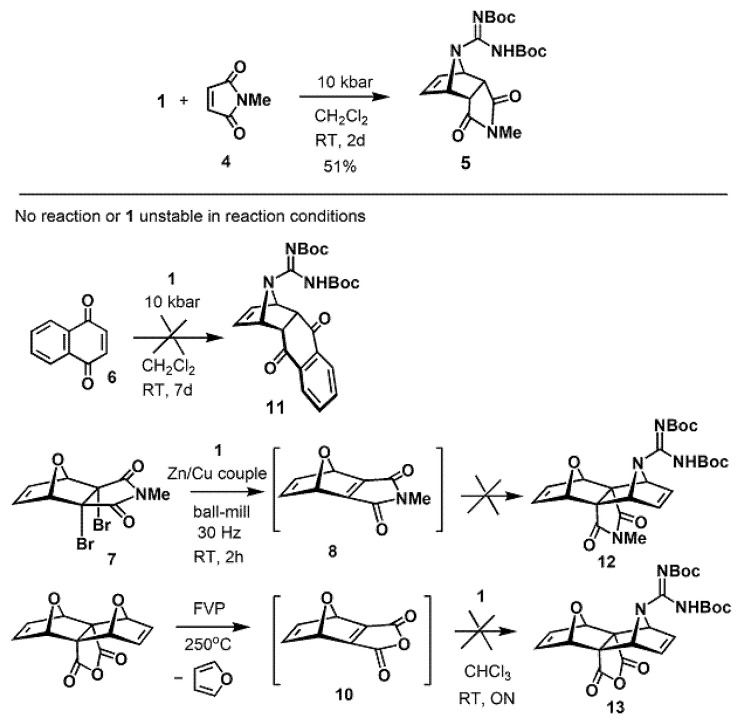
Cycloaddition reactions of pyrrole **1**.

The 1-(*N*′,*N*′-Di-Boc)pyrrole carboxamidine **1** was prepared according to the literature starting from 3-pyrroline [15,16]. Pyrrole **1** showed poor reactivity in cycloaddition reactions when acting as 1,3-diene (Scheme 1). For instance, the thermal reaction of **1** with *N*-methylmaleimide did not provide the expected cycloadduct **5**. In order to increase its reactivity, an extremely high-pressure technique was employed [17]. The pressurization at 10 kbar, for 2 days, in dichloromethane resulted in the formation of **5** in 51% yield. It was found that product **5** was unstable in CHCl_3_ solution (also in solid state) and quickly cycloreverses back to reactants. This behavior could explain the failure of thermal conditions. An alternative way to increase dienes’ reactivity is to employ more reactive dienophiles. Thermal and high-pressure reactions with naphthoquinone did not provide conclusive evidence for the formation of a cycloadduct. Equally unsuccessful were reactions of mechanochemically solid state in-situ-generated imide **8** [18] in a ball mill, due to harsh conditions for the guanidine moiety of **1**. Reaction with related anhydride **10** [19] also provided a complex reaction mixture.

### 2.2. Synthesis of Isoindoles and Their Reactivity

**Scheme 2:**
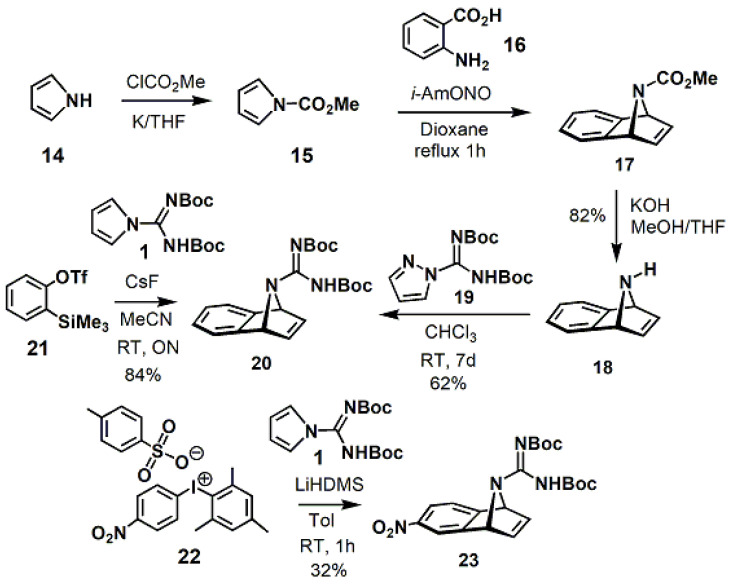
Synthesis of isoindole precursors **20** and **23**.

Further increase in the reactivity of pyrroles could be achieved by the addition of a benzene ring, i.e., to use isoindole derivatives as dienes. Synthesis of isoindole precursor **20** in four reaction steps is shown in Scheme 2. It follows the already established synthesis of 7-azabenzonorbornadiene **18**, and subsequent guanylation with *N,N*′-Di-Boc-1H-pyrazole-1-carboxamidine provided **20** in 62% yield. Alternatively, **20** could be prepared by in-situ-generated benzyne cycloaddition with pyrrole **1** (84%). Preparation of a nitro derivative of **20** was achieved by in-situ generation of 4-nitro benzyne from iodonium salt **22** [20] and its reaction with pyrrole **1**, which provided cycloadduct **23** in 32% yield. These reactions show that the reactivity of pyrrole-1-carboxamidine **1** could be increased by the presence of a highly reactive dienophile such as arynes.

**Scheme 3:**
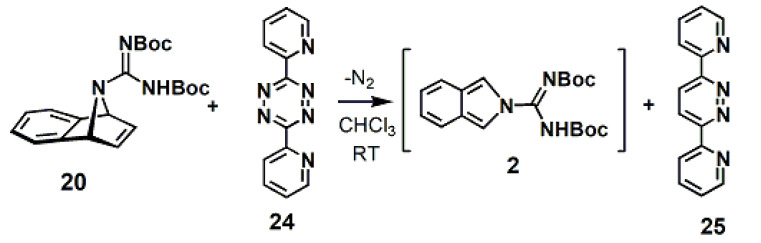
Generation of isoindole **2** using tetrazine method.

**Figure 2:**
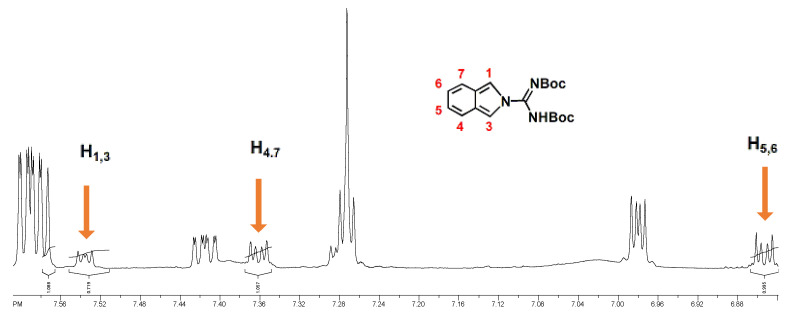
Section of ^1^H NMR spectra (in CDCl_3_) with assigned peaks of isoindole **2**.

Warrener’s cycloaddition/elimination/cycloreversion method employing bis(2-pyridyl)-*sym*-1,2,4,5-tetrazine **24** [21,22] was used for the generation of isoindole **2** (Scheme 3). The formation of **2** was confirmed by ^1^H NMR spectroscopy, by spectrum recorded 30 min after the addition of **24** to a solution of **20** in an NMR tube (Figure 2). The most characteristic signals which indicate the presence of **2** are a singlet of H_1,3_ appearing at δ 7.57, whereas aromatic multiplets of H_4,7_ and H_5,6_ are found at δ 7.36 and 6.85. However, trapping experiments offer indirect but more solid evidence of its formation.

**Scheme 4:**
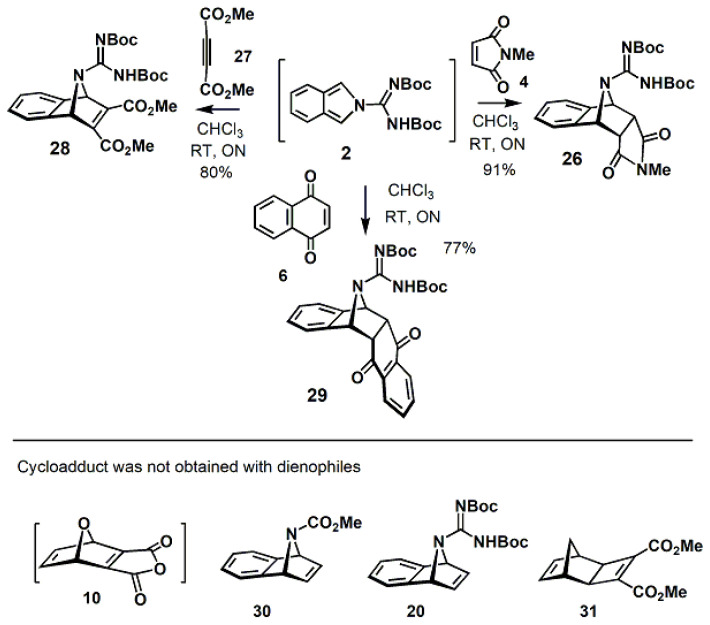
Cycloaddition reactions of isoindole **2**.

Scheme 4 summarizes the cycloaddition properties of isoindole-2-carboxamidine **2**. When **2** was generated in the presence of dienophiles *N*-methylmaleimide, dimethylacetylenedicarboxylate (DMAD), and benzoquinone, corresponding cycloadducts **26**, **28**, and **29** were obtained (in 91, 80 and 77% yields, respectively). In variance, norbornenes **10**, **20**, **30,** and **31** did not react or afforded intractable mixtures, regardless of reaction conditions (thermal or high pressure). The *endo-*adduct **26** was solely formed, as shown by the single methyl resonance at δ 2.28 in the ^1^H NMR spectrum (see Supplementary Materials), and the *endo-*configuration is proven by the shielding of the *N*-methyl protons by the ring current effect of the aromatic ring [22]. Furthermore, the *exo-*protons are multiplets, is characteristic of the *endo-*adducts of isoindoles [23,24]. This *endo-*stereospecificity is similar to *N*-benzyl-isoindole cycloaddition [25] and in variance with maleic anhydride reactions of isoindoles where *exo/endo* mixtures were formed, [24,26] whereas the outcome of cycloadditions of 2-substituted isoindoles with tolyl-maleimide was not specified [27].

An interesting feature of the ^1^H NMR spectra of cycloaddition products **26** and **28** is the broadness of bridgehead signals at 20 °C. Recording the spectra at 50 °C led to the sharpening of the signal, whereas cooling down to 5 °C gives two sets of bridgehead signals, which are associated with nitrogen inversion [28]. The *N*-inversion barrier in **26** is estimated to be low, 13.5 kcal mol^−1^ in deuterated chloroform. A similar broadness of bridgehead protons was observed for pyrrole cycloadduct **5**; however, this adduct is thermally unstable and quickly cycloreverses.

**Scheme 5:**
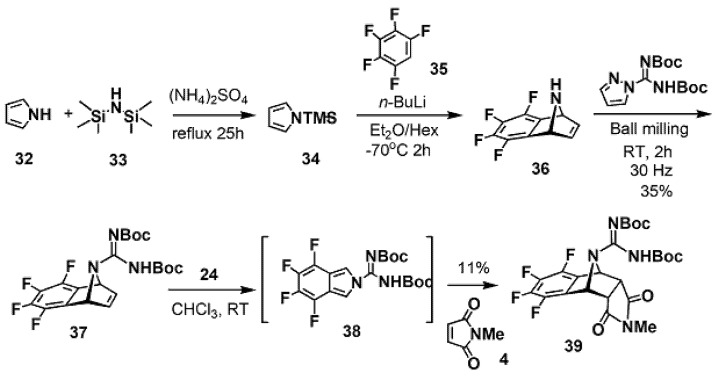
Synthesis and reactions of tetrafluoro isoindole **38**.

In continuation, the electronics of isoindoles were altered by fluorine substituents on the aromatic ring and positioning of the guanidine functionality. Tetrafluoro isoindole precursor **37** was prepared in 35% yield by mechanochemical guanylation [29] of the known 7-azabenzonorbornadiene **36** [30,31] (Scheme 5). It was found that fluorine substitution did not have noticeable effects on the cycloaddition reactivity of isoindole. When tetrafluoroisoindole **38** was trapped in a tetrazine reaction with **37**, the *endo*-cycloadduct **39** was obtained in 11% yield, while, similarly to isoindole **2**, tetrafluoro derivative **38** also did not react with its precursor **37**.

**Scheme 6:**
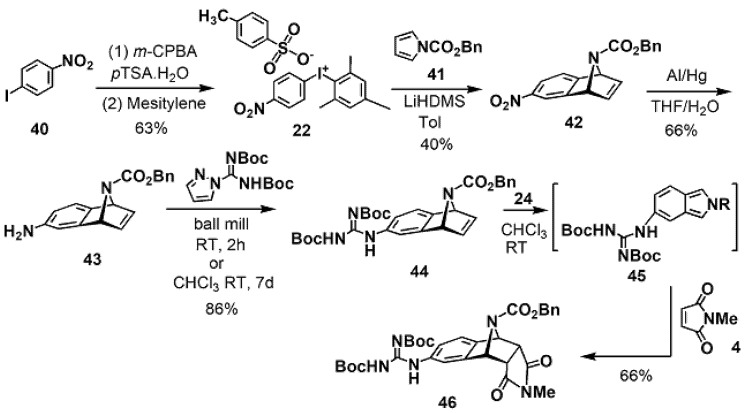
Synthesis and reactions of isoindole **45**.

Until now, isoindoles substituted on an aromatic ring with the nitrogen atom have been known only with the nitro group, [32,33,34,35] and we prepared the first example of a guanidine aromatic-ring-substituted isoindole. The synthetic route for the introduction of guanidine functionality at position 5 of the isoindole ring in **45** is depicted in Scheme 6. In-situ-generated 4-nitro-benzyne was reacted with 1-benzyloxycarbonyl pyrrole **41** to afford the known cycloadduct **42** [23]. The nitro group was reduced by Al/Hg and the amine **43** was obtained in 66% yield. Guanylation in solution led to the formation of isoindole precursor **44** in 86% yield. This compound was treated with tetrazine **24** in chloroform and intermediate isoindole **45** was trapped as *N*-methylmaleimide cycloadduct **46** (66%). The change in the position of guanidine functionality and *N*-CBz substitution did not increase the cycloaddition reactivity of isoindole. Analogously to isoindoles **5** and **38**, in the case of **45**, a reaction with **44** as a dienophile was not observed. These results indicate the similar cycloaddition reactivity of all three investigated guanidine isoindoles.

**Figure 3:**
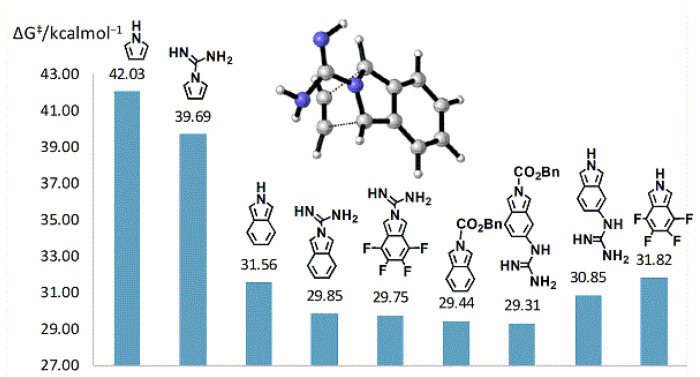
Activation-free energies of the Diels–Alder reaction of pyrrole and isoindoles with acetylene and optimized transition state structure of the reaction with isoindole-2-carboxamidine as calculated by M062X/6-311+G**.

Previous density functional theory (DFT) calculations B3LYP/6-31G(d) predict that activation energies (*E*_a_) for reactions of pyrrole and isoindole-2-carboxamidine with DMAD are 32.37 and 23.17 kcal mol^−1^, respectively, indicating that the amidine substitution decreases *E*_a_ by 4–5 kcal mol^−1^ in comparison to parent unsubstituted dienes, whereas Boc protection of amidinopyrrole causes a further drop in *E*_a_ by 2.5 kcal mol^−1^. Now, these theoretical predictions are supplemented with the M062X/6-311+G** calculations [23] of the reaction of acetylene with pyrrole and isoindoles. All located transition states possess structures resembling the synchronous concerted mechanism of Diels–Alder reactions, such as the one illustrated in Figure 3. Computed activation-free energies (ΔG^⧧^ values) are given in Figure 3 and reveal similar predictions to the previously obtained B3LYP calculations. Firstly, *N*-substitution with amidine lowers ΔG^⧧^ by 1.5–2.3 kcal mol^−1^. The largest difference in ΔG^⧧^ values was obtained when pyrrole was fused with a benzene ring in isoindoles, which is in qualitative accordance with published AM1 results [23]. The position of an amidine (guanidine) substituent and the addition of fluorine atoms has only a marginal effect on the ΔG^⧧^ values, with differences in the reactivity of three experimentally studied isoindoles within 0.54 kcal mol^−1^. These predictions are in full accordance with almost identical experimentally observed reactivities of three isoindoles.

## 3. Materials and Methods

### 3.1. General

Solvents and chemicals were obtained from Tokyo Chemical Industry (Tokyo, Japan) and Sigma Aldrich (Burlington, VT, USA). Kemika (Zagreb, Croatia), Sigma Aldrich, and VWR Chemicals (Radnor, PA, USA) supplied the solvents, which were used without further purification, unless otherwise stated. The NMR spectra were recorded on Bruker Avance 300 MHz and Bruker Avance 600 MHz spectrometers in deuterated solvents. Chemical shifts (δ) are given in ppm using tetramethylsilane (TMS) as an internal standard, whereas coupling constants (*J*) are expressed in Hertz (Hz). The following abbreviations were used to describe multiplicity in the ^1^H spectra: (s) singlet; (d) doublet; (dd) doublet of doublets; (t) triplet; (m) multiplet; (brs) broad signal. Fourier Transform Infrared Attenuated Total Reflection PerkinElmer UATR Two Spectrometer (range 400–4000 cm^−1^) was used to record infrared spectra (FTIR-ATR). Milling reactions were carried out in Retsch MM400 vibrational mill (frequency 30 Hz), using stainless steel (SS) vials (10 mL) and one 12 mm size SS milling ball. High-pressure reactions were performed in Teflon vials (V = 1.5 mL) using a high-pressure-piston cylinder apparatus (Unipress, Polish Academy of Sciences), and pentane as a pressure-transmitting liquid. Thin-layer chromatography (TLC) was performed on silica-gel plates (silica gel 60 F_254_, Merck), whereas silica gel (Silica gel 60, 0.063–0.200 mm, Merck, Darmstadt, Germany) was used for column chromatography. High-resolution mass spectra (HRMS) were recorded on Agilent 6550 Series Accurate-Mass-Quadrupole Time-of-Flight (Q-TOF) Agilent 1290 Infinity II instrument.

### 3.2. Synthesis of Cycloadduct ***5***

Pyrrole **1** (20 mg, 0.065 mmol) and *N*-methylmaleimide (11 mg, 0.098 mmol) were dissolved in CH_2_Cl_2_ (1 mL) and the solution was subjected to high pressure at 10 kbar for 48 h at room temperature. The reaction mixture was evaporated and purified by column chromatography, starting with petroleum ether/EtOAc mixture from 5:1 to 2.5:1. Two fractions were isolated, pyrrole **1** (11 mg) and product **5** as colorless solid (14 mg, 51%).

^1^H NMR (CDCl_3_), δ/ppm: 1.49 (s, 18H, *t*-Bu), 2.84 (s, 3H, NCH_3_), 3.69 (dd, 1H, *J* = 3.3, 1.5 Hz, *exo*-H), 5.31 (brs, 2H, N bridge), 6.39 (brs, 2H, C=CH), 10.60 (brs, 1H, NH),

FTIR-ATR ν_max_/cm^−1^: 2979, 1700 (C=O), 1600 (C=O), 1275, 1121.

### 3.3. Synthesis of ***20***

Benzonorbornadiene **18** (25 mg, 0.175 mmol) and *N,N*′-Di-Boc-1H-pyrazole-1-carboxamidine (49 mg, 0.158 mmol) were dissolved in CHCl_3_ (1 mL) and stirred at room temperature for 7 days. The reaction mixture was purified by radial chromatography using CH_2_Cl_2_. Product **20** was isolated as a white solid (42 mg, 62%).

m.p. 158–160 °C,

^1^H NMR (CDCl_3_), δ/ppm: 1.49 (s, 9H, *t*-Bu), 1.50 (s, 9H, *t*-Bu), 5.84 (brs, 2H, N bridge), 6.98 (dd, 1H, *J* = 5.2, 3.2 Hz, Ar), 7.02 (d, 2H, *J* = 2.6 Hz, C=CH), 7.27 (dd, 1H, *J* = 5.2, 3.2 Hz, Ar), 10.63 (brs, 1H, NH),

^13^C NMR (CDCl_3_), δ/ppm: 28.1 (*t*-Bu), 28.2 *(t*-Bu), 65.8 (N bridge), 68.2 (N bridge), 79.8, 82.2, 120.9 (C=C), 125.3, 144.1, 147.5, 150.2 (C=O), 151.2 (C=N), 162.8 (C=O),

FTIR-ATR ν_max_/cm^−1^: 2977, 1756 (C=O), 1636 (C=O),

HRMS-MALDI found: 386.2087, calculated for C_21_H_28_N_3_O_4_ [MH]^+^: 386.2080.

### 3.4. Synthesis of Cycloadduct ***23***

Under argon, dry toluene (1 mL) was added to pyrrole **1** (20 mg, 0.064 mmol) and iodonium salt **22** (35 mg, 0.064 mmol). LiHDMS solution in toluene (64 μL, 0.064 mmol, 1 M) was added dropwise and the resulting mixture was stirred for 1 h at room temperature. The reaction was quenched with saturated NH_4_Cl solution (5 mL) and extracted with EtOAc (3 × 10 mL), and combined extracts were dried with Na_2_SO_4_ and evaporated. The crude mixture was purified by radial chromatography using petroleum ether/diethyl ether 5:1 and gradually increasing polarity to 1:1. Product **23** was isolated as a yellow solid (9 mg, 32%).

m.p. 88–90 °C,

^1^H NMR (CDCl_3_), δ/ppm: 1.50 (s, 18H, *t*-Bu), 5.93 (brs, 2H, N bridge), 7.05 (brs, 2H, C=CH), 7.39 (d, 1H, *J* = 7.9 Hz, Ar), 7.99 (dd, 1H, *J* = 7.9, 1.9 Hz, Ar), 8.08 (d, 1H, *J* = 1.9 Hz, Ar), 10.68 (brs, 1H, NH),

^13^C NMR (CDCl_3_), δ/ppm δ/ppm: 28.0 (*t*-Bu), 28.1 (*t*-Bu), 80.3 (N bridge), 82.5, 82.6, 82.7 (N bridge), 122.4 (C=C), 145.9, 149.6, 149.8, 150.0, 150.1, 151.9, 155.0 (C=N), 162.5 (C=O), 162.6 (C=O),

FTIR-ATR ν_max_/cm^−1^: 2979, 1754 (C=O), 1683 (C=O),

HRMS-MALDI found: 431.1942, calculated for C_21_H_27_N_4_O_6_ [MH]^+^: 431.1931.

### 3.5. Synthesis of Cycloadduct ***26***

Isoindole precursor **20** (98 mg, 0.25 mmol), bis(2-pyridyl)-*sym*-1,2,4,5-tetrazine **24** (59 mg, 0.25 mmol) and *N*-methylmaleimide (28 mg, 0.25 mmol) were dissolved in CHCl_3_ (2.5 mL) and stirred overnight at room temperature. The reaction mixture was purified by radial chromatography using CH_2_Cl_2_/MeOH mixture 99:1. Cycloadduct **26** was isolated as a white solid (107 mg, 91%).

m.p. 120–122 °C,

^1^H NMR (CDCl_3_), δ/ppm: 1.50 (s, 18H, *t-*Bu), 2.28 (s, 3H, NCH_3_), 3.88 (d, 1H, *J* = 1.9 Hz, *exo-*H), 5.76 (brs, 2H, N bridge), 7.21 (dd, 1H, *J* = 5.5, 3.1 Hz, Ar), 7.28 (dd, 1H, *J* = 5.5, 3.1 Hz, Ar), 10.60 (brs, 1H, NH),

^13^C NMR (CDCl_3_), δ/ppm: 23.9 (exo-H), 28.1 (*t*-Bu), 47.2 (NCH_3_), 62.6 (N bridge), 80.4, 82.8, 121.6, 126.0, 139.3, 150.2 (C=O), 152.6, (C=O), 162.6 (C=N), 174.5 (C=O),

FTIR-ATR ν_max_/cm^−1^: 2975, 1751 (C=O), 1704 (C=O), 1650 (C=O),

HRMS-MALDI found: 471.2255, calculated for C_24_H_31_N_4_O_6_ [MH]+: 471.2244.

### 3.6. Synthesis of Cycloadduct ***28***

Isoindole precursor **20** (50 mg, 0.13 mmol), bis(2-pyridyl)-*sym*-1,2,4,5-tetrazine **24** (31 mg, 0.13 mmol), and DMAD (8.0 µL, 0.065 mmol) were dissolved in CHCl_3_ (1 mL) and stirred overnight at room temperature. The reaction mixture was purified by radial chromatography using CH_2_Cl_2_. Cycloadduct **28** was isolated as an off-white solid (52 mg, 80%).

m.p. 70–72 °C,

^1^H NMR (CDCl_3_), δ/ppm: 1.49 (s, 18H, *t*-Bu), 3.80 (s, 6H, OCH_3_), 6.12 (brs, 2H, N bridge), 7.07 (dd, 1H, *J* = 5.4, 3.1 Hz, Ar), 7.43 (dd, 1H, *J* = 5.4, 3.1 Hz, Ar), 10.61 (brs, 1H, NH),

FTIR-ATR ν_max_/cm^−1^: 2979, 1755 (C=O), 1722 (C=O),

^13^C NMR (CDCl_3_), δ/ppm: 28.1 (*t*-Bu), 52.4 (N bridge), 109.8 (C=C), 122.2, 126.2, 128.9, 139.1, 142.7 (C=O), 145.0 (C=O), 162.4 (C=O), 162.7 (C=N),

HRMS-MALDI found: 502.2197, calculated for C25H_32_N_3_O_8_ [MH]^+^: 502.2189.

### 3.7. Synthesis of Cycloadduct ***29***

Isoindole precursor **20** (50 mg, 0.13 mmol), bis(2-pyridyl)-*sym*-1,2,4,5-tetrazine **24** (31 mg, 0.13 mmol) and naphthoquinone monohydrate (23 mg, 0.13 mmol) were dissolved in CHCl_3_ (1 mL) and stirred overnight at room temperature. The reaction mixture was purified by radial chromatography using CH_2_Cl_2_. Cycloadduct **29** was isolated as a brown solid (52 mg, 77%).

m.p. 95–97 °C,

^1^H NMR (CDCl_3_), δ/ppm: 1.52 (s, 18H, *t*-Bu), 4.01 (s, 2H, *exo-*H), 5.92 (brs, 2H, N bridge), 6.87 (dd, 1H, *J* = 6.9, 3.2 Hz, Ar), 7.10 (dd, 1H, *J* = 6.9, 3.2 Hz, Ar), 7.52 (dd, 1H, *J* = 5.9, 3.3 Hz, Ar), 7.77 (dd, 1H, *J* = 5.9, 3.3 Hz, Ar), 10.58 (brs, 1H, NH),

^13^C NMR (CDCl_3_), δ/ppm: 28.2 (*t*-Bu), 49.6 (exo-H), 66.8 (N bridge), 80.2, 82.8, 121.8, 126.6, 127.4, 134.1, 134.6, 140.6, 150.2 (C=O), 152.3, (C=O), 162.6 (C=N), 194.4 (C=O),

FTIR-ATR ν_max_/cm^−1^: 2980, 1732 (C=O), 1677 (C=O),

HRMS-MALDI found: 518.2295, calculated for C_29_H_32_N_3_O_6_ [MH]^+^: 518.2291.

### 3.8. Synthesis of ***37***

Tetrafluoroazabenzonorbornadiene **36** (215 mg, 1.0 mmol) and *N,N*′-Di-Boc-1H-pyrazole-1-carboxamidine (310 mg, 1.0 mmol) were grinded in a ball mill for 2 h. The reaction mixture was purified by radial chromatography using CH_2_Cl_2_/hexane mixture 70:30. Cycloadduct **37** was isolated as a brown solid (158 mg, 35%).

m.p. 84–86 °C,

^1^H NMR (CDCl_3_), δ/ppm: 1.50 (s, 18H, *t-*Bu), 6.10 (brs, 2H, N bridge), 7.06 (brs, 2H, C=CH), 10.61 (brs, 1H, NH),

^13^C NMR (CDCl_3_), δ/ppm: 28.1 (*t*-Bu), 62.9 (N bridge), 65.4(N bridge), 80.5, 82.8, 129.2 (d, ^4^*J*_CF_ = 17 Hz, C=C), 138.0 (t, ^2^*J*_CF_ = 16.1 Hz, Ar), 139.6 (t, ^2^*J*_CF_ = 16.1 Hz, Ar), 141.2 (m, Ar), 144.1 (m, Ar), 149.9 (C=O), 151.6 (C=O), 162.4 (C=N),

FTIR-ATR ν_max_/cm^−1^: 2981, 1760 (C=O), 1653 (C=O),

HRMS-MALDI found: 458.1716, calculated for C_21_H_24_F_4_N_3_O_4_ [MH]^+^: 458.1703.

### 3.9. Synthesis of Cycloadduct ***39***

Isoindole precursor **38** (15 mg, 0.033 mmol), bis(2-pyridyl)-*sym*-1,2,4,5-tetrazine **24** (8 mg, 0.033 mmol) and *N*-methylmaleimide (4 mg, 0.036 mmol) were dissolved in CHCl_3_ (0.5 mL) and stirred overnight at 60 °C. The reaction mixture was purified by radial chromatography using CH_2_Cl_2_. Cycloadduct **39** was isolated as a white solid (2 mg, 11%).

m.p. 108–110 °C,

^1^H NMR (CDCl_3_), δ/ppm: 1.49 (s, 9H, *t-*Bu), 1.52 (s, 9H, *t-*Bu), 2.56 (s, 3H, NCH_3_), 3.96 (dd, 2H, *J* = 3.8, 1.8 Hz, *exo-*H), 5.99 (brs, 2H, N bridge), 10.59 (brs, 1H, NH),

^13^C NMR (CDCl_3_), δ/ppm: 24.6 (NCH_3_), 28.0 (*t*-Bu), 47.1, 60.0 (N bridge), 65.2 (N bridge), 81.1, 83.5, (140.3, 146.6, 149.9), 152.5 (C=O), 162.1 (C=N), 173.2 (C=O),

FTIR-ATR ν_max_/cm^−1^: 2978, 1741 (C=O), 1709 (C=O), 1662 (C=O),

HRMS-MALDI found: 543.1874, calculated for C_24_H_27_F_4_N_4_O_6_ [MH]^+^: 543.1867.

### 3.10. Synthesis of ***42***

Under argon, dry toluene (11 mL) was added to Cbz-pyrrole **41** (200 mg, 1.0 mmol) and iodonium salt **22** (539 mg, 1.0 mmol). LiHDMS solution in toluene (1.0 mL, 1.0 mmol, 1 M) was added dropwise and the resulting mixture was stirred for 1 h at room temperature. The reaction was quenched with saturated NH_4_Cl solution (40 mL) and extracted with EtOAc (3x30 mL); combined extracts were dried with Na_2_SO_4_ and evaporated. The crude mixture was purified by radial chromatography using CH_2_Cl_2_ and gradually increasing polarity with MeOH. Product **42** was isolated as a viscous yellow oil (128 mg, 40%).

^1^H NMR (CDCl_3_), δ/ppm: 5.06 (s, 2H, CH_2_), 5.67 (brs, 2H, N bridge), 7.02 (d, 2H, *J* = 10.1 Hz, C=CH), 7.23–7.25 (m, 2H, Ar), 7.30–7.37 (m, 4H, Ar), 7.95 (dd, 1H, *J* = 7.9, 1.8 Hz, Ar), 8.03 (brs, 1H, Ar),

^13^C NMR (CDCl_3_), δ/ppm: 66.1 (N bridge), 67.0 (OCH_2_), 67.6 (N bridge), 122.2 (C=C), 127.9, 128.2, 128.3, 128.6, 128.7, 128.8, 128.9, 129.0, 135.8, 145.7, 155.0 (C=O),

FTIR-ATR ν_max_/cm^−1^: 2955, 1708 (C=O), 1517 (N-O), 1323 (N-O),

HRMS-MALDI found: 323.1038, calculated for C_18_H_15_N_2_O_4_ [MH]^+^: 323.1032.

### 3.11. Synthesis of ***43***

Azabenzonorbornadiene **42** (89 mg, 0.28 mmol) was dissolved in THF/H_2_O mixture (40 mL, 10% H_2_O) and heated to 60 °C. Aluminium amalgam was prepared by immersing aluminium foil (400 mg) in a solution of HgCl_2_ (500 mg) in water (50 mL) for 1 min, followed by washing in ethanol (50 mL) and diethyl ether (50 mL). Amalgam was added to the solution and the mixture was continuously heated for 1 h, filtered through Celite and washed with THF. The filtrate was evaporated and purified by radial chromatography using CH_2_Cl_2_ and gradually increasing polarity with MeOH. Product **43** was isolated as a viscous brown oil (53 mg, 66%).

^1^H NMR (CDCl_3_), δ/ppm: 3.56 (brs, 2H, NH_2_), 5.06 (s, 2H, CH_2_), 5.49 (d, 2H, *J* = 6.6 Hz, N bridge), 6.22 (dd, 1H, *J* = 7.6, 2.0 Hz, Ar), 6.69 (brs, 1H), 6.85–6.95 (m, 3H, C=C, Ar), 7.24–7.33 (m, 5H, Ar),

^13^C NMR (CDCl_3_), δ/ppm: 65.8 (N bridge), 67.2(N bridge), 67.3 (OCH_2_),112.7 (C=C), 121.4 (C=C), 127.7, 127.8, 128.1, 128.2, 128.5, 128.6, 130.1, 130.2, 136.1, 136.2, 155.2 (C=O),

FTIR-ATR ν_max_/cm^−1^: 3361 (N-H_2_), 1699 (C=O),

HRMS-MALDI found: 293.1292, calculated for C_18_H_17_N_2_O_2_ [MH]^+^: 293.1290.

### 3.12. Synthesis of Guanidine ***44***

Azabenzonorbornadiene **43** (167 mg, 0.572 mmol) and *N,N*′-Di-Boc-1H-pyrazole-1-carboxamidine (177 mg, 0.572 mmol) were dissolved in CHCl_3_ (5 mL) and stirred at room temperature for 2 days. The crude mixture was purified by radial chromatography using petroleum ether and gradually increasing polarity with CH_2_Cl_2_. Guanidine **44** was isolated as a viscous yellow oil (262 mg, 86%).

^1^H NMR (CDCl_3_), δ/ppm: 1.50 (s, 9H, *t*-Bu), 1.53 (s, 9H, *t*-Bu), 5.06 (d, 2H, *J* = 13.8 Hz, CH_2_), 5.56 (d, 2H, *J* = 16.1 Hz, N bridge), 6.92 (brs, 1H, C=CH), 6.97 (brs, 1H, C=CH), 7.05–7.21 (m, 3H, Ar), 7.26–7.34 (m, 5H, Ar), 10.29 (brs, 1H; NH), 11.64 (brs, 1H, NH),

^13^C NMR (CDCl_3_), δ/ppm: 28.1 (*t*-Bu), 28.2 (*t*-Bu), 65.9 (N bridge), 66.4 (N bridge), 67.2 (OCH_2_), 116.5 (C=C), 118.3, 120.9 (C=C), 127.8, 128.0, 128.5, 129.0, 133.9, 136.3, 142.8, 144.3, 149.2, 153.3 (C=O), 153.6 (C=O), 155.1 (C=N), 163.5 (C=O),

FTIR-ATR ν_max_/cm^−1^: 2978, 1713 (C=O),

HRMS-MALDI found: 535.2572, calculated for C_29_H_35_N_4_O_6_ [MH]^+^: 535.2557.

### 3.13. Synthesis of Cycloadduct ***46***

Isoindole precursor **44** (15 mg, 0.028 mmol), bis(2-pyridyl)-*sym*-1,2,4,5-tetrazine **24** (7.0 mg, 0.029 mmol) and *N*-methylmaleimide (3.0 mg, 0.027 mmol) were dissolved in CHCl_3_ (0.5 mL) and stirred overnight at room temperature. The reaction mixture was purified by radial chromatography using CH_2_Cl_2_. Cycloadduct **46** was isolated as a white solid (11 mg, 66%).

m.p. 100–102 °C,

^1^H NMR (CDCl_3_), δ/ppm: 1.49 (s, 9H, *t-*Bu), 1.53 (s, 9H, *t*-Bu), 2.36 (s, 3H, NCH_3_), 3.69 (brs, 2H, *exo-*H), 5.08 (brs, 2H, CH_2_), 5.52 (d, 1H, *J* = 4.4 Hz, N bridge), 5.56 (d, 1H, *J* = 4.4 Hz, N bridge), 7.21 (d, 1H, *J* = 8.0 Hz, Ar), 7.31–7.38 (m, 5H, Ar), 7.49 (brs, 2H, Ar), 10.29 (brs, 1H, NH), 11.58 (brs, 1H, NH),

^13^C NMR (CDCl_3_), δ/ppm: 28.1 (*t*-Bu), 28.2 (*t*-Bu), 45.6 (*exo-*H), 46.6 (*exo-*H), 62.4 (CH_2_), 62.8 (N bridge), 67.8 (N bridge), 79.8, 83.9, 116.2, 122.1, 128.1, 128.4, 128.5, 128.6, 135.7. 135.9, 136.8. 140.5, 153.3 (C=O), 153.6 (C=O), 154.6 (C=O), 163.4 (C=N), 174.1 (C=O), 174.2 (C=O),

FTIR-ATR ν_max_/cm^−1^: 2980, 1704 (C=O), 1634 (C=O),

HRMS-MALDI found: 620.2730, calculated for C_32_H_38_N_5_O_8_ [MH]^+^: 620.2720.

## 4. Conclusions

Novel isoindoles possessing guanidine substituents were synthesized and their cycloaddition reactivity was explored. These show higher reactivity than the corresponding pyrrole-1-carboxamidine and lead to the formation of polycyclic structures which incorporate guanidine functionality into the 7-azanobornene skeleton. New isoindoles were reactive towards dienophiles possessing electron-withdrawing groups (*N*-methylmaleimide, DMAD, naphthoquinone) and highly reactive arynes, whereas reactions with 7-azabenzonorbornadiene did not occur. In addition, 2H-isoindole-2-carboxamidine, its tetrafluoro counterpart and 4-guanidino isoindole showed similar reactivity. Experimentally observed reactivities are in good accord with theoretical predictions obtained at the M062X/6-311+G** level. Only small (about 0.5 kcal mol^−1^) differences in energy barriers for cycloaddition reactions of isoindoles were predicted.

## Data Availability

Data are contained within the article and the Supplementary Materials.

## Supplementary Materials

The following supporting information can be downloaded at: https://www.mdpi.com/article/10.3390/molecules27248954/s1, ^1^H and ^13^C NMR and IR spectra for the synthesized compounds.
