# Supplementary material for: N,N′-Di-Boc-2H-Isoindole-2-carboxamidine—First Guanidine-Substituted Isoindole
Source: Molecules. 2022 Dec 15;27(24):8954. doi: 10.3390/molecules27248954 (PMC9781607; doi:10.3390/molecules27248954)
Supplement: Supplementary file 1 [file molecules-27-08954-s001.zip › molecules-2105651-SI.pdf]

SUPPORTING INFORMATION

N,N′-Di-Boc-2H-Isoindole-2-Carboxamidine—First Guanidine-Substituted Isoindole

NMR and IR spectra

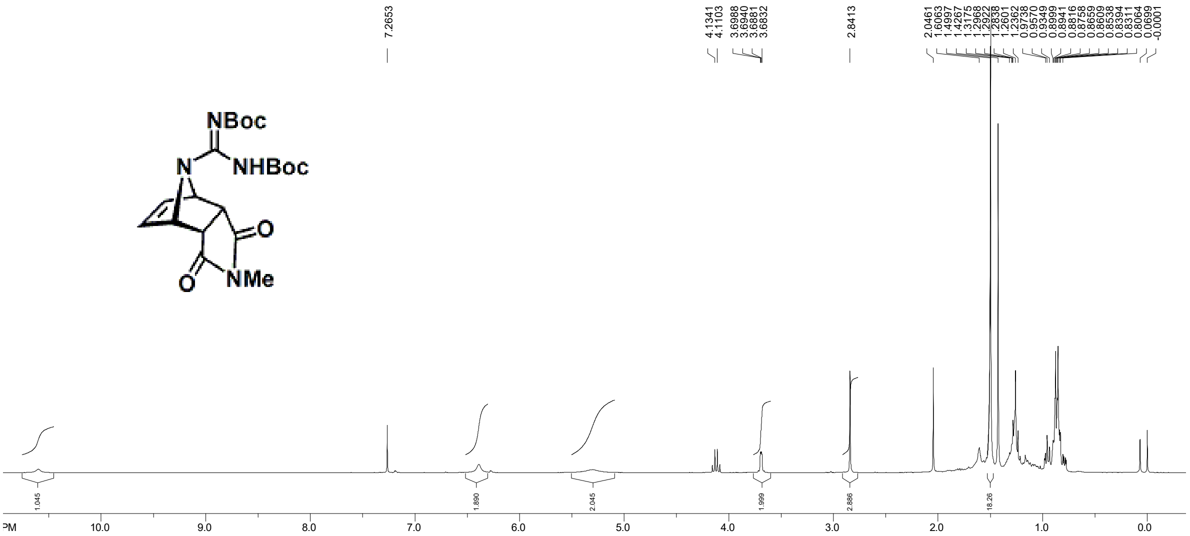

Figure S1. ^{1}H NMR (300 MHz, $CDCl_3$) spectrum of cycloadduct **5.**

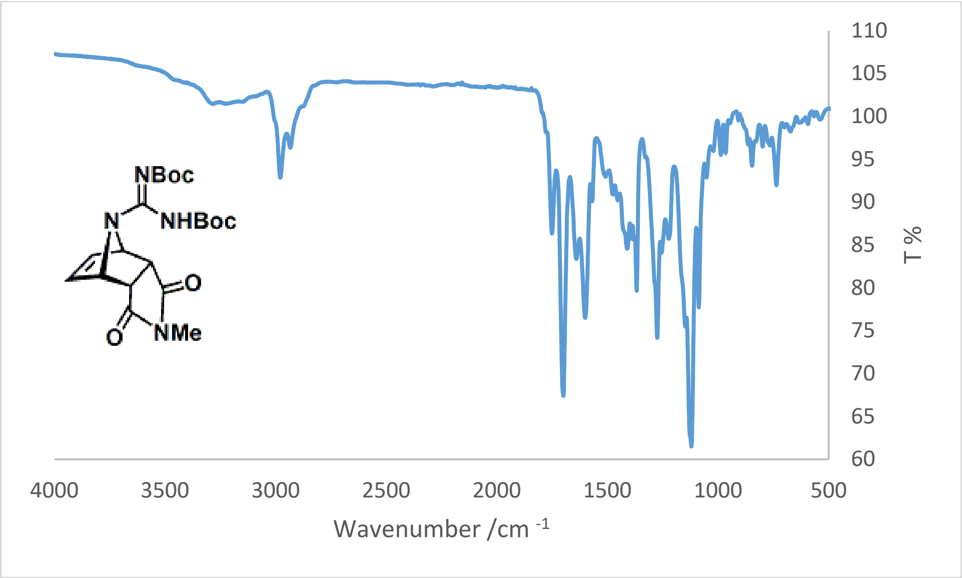

Figure S2. FTIR-ATR spectrum of cycloadduct **5.**

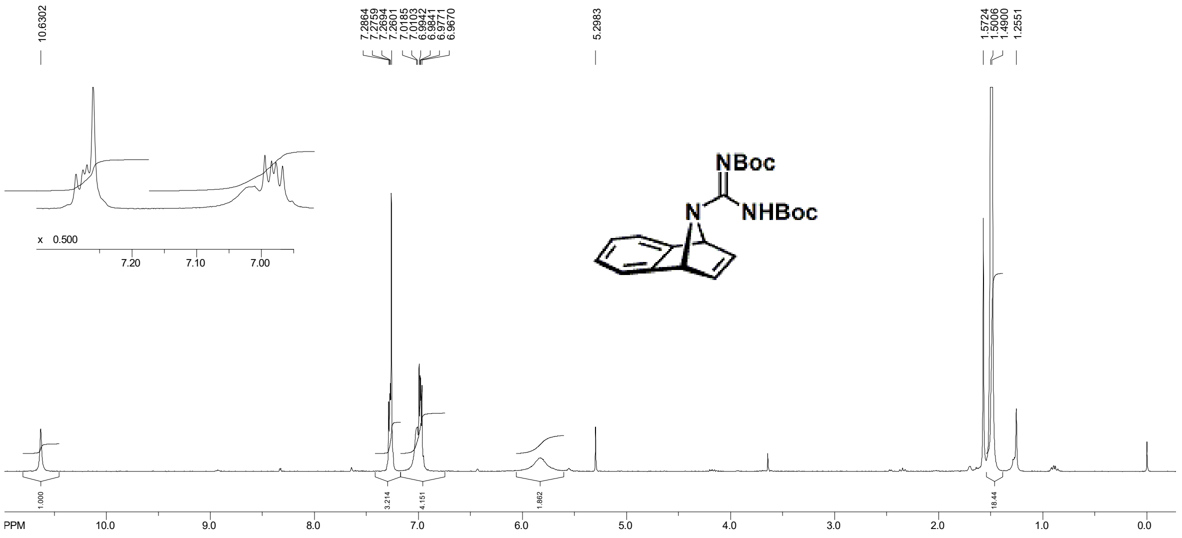

Figure S3. ^{1}H NMR (300 MHz, $CDCl_3$) spectrum of guanidine **20.**

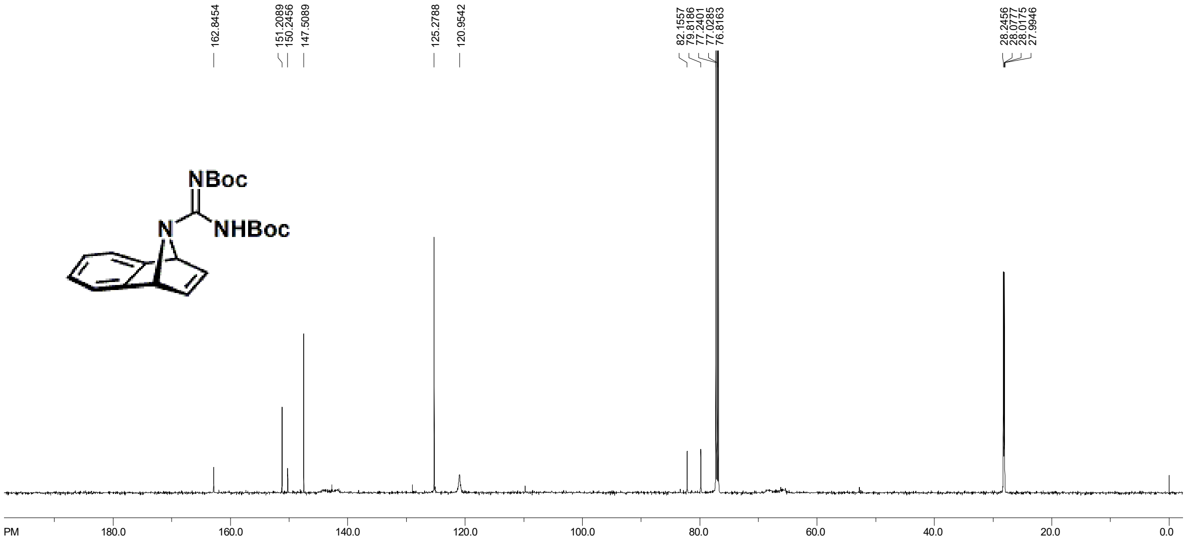

Figure S4. ^{13}C NMR (300 MHz, $CDCl_3$) spectrum of guanidine **20.**

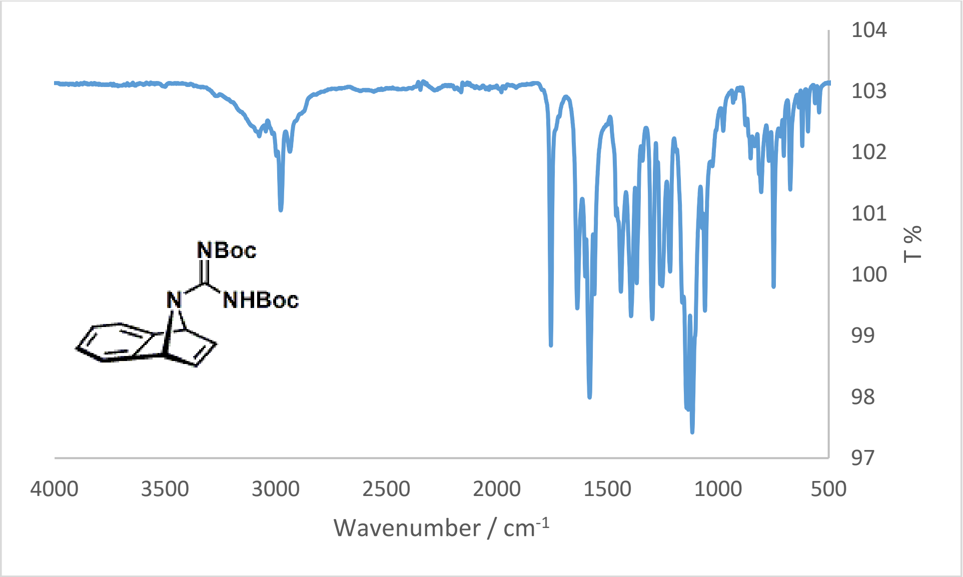

Figure S5. FTIR-ATR spectrum of guanidine **20.**

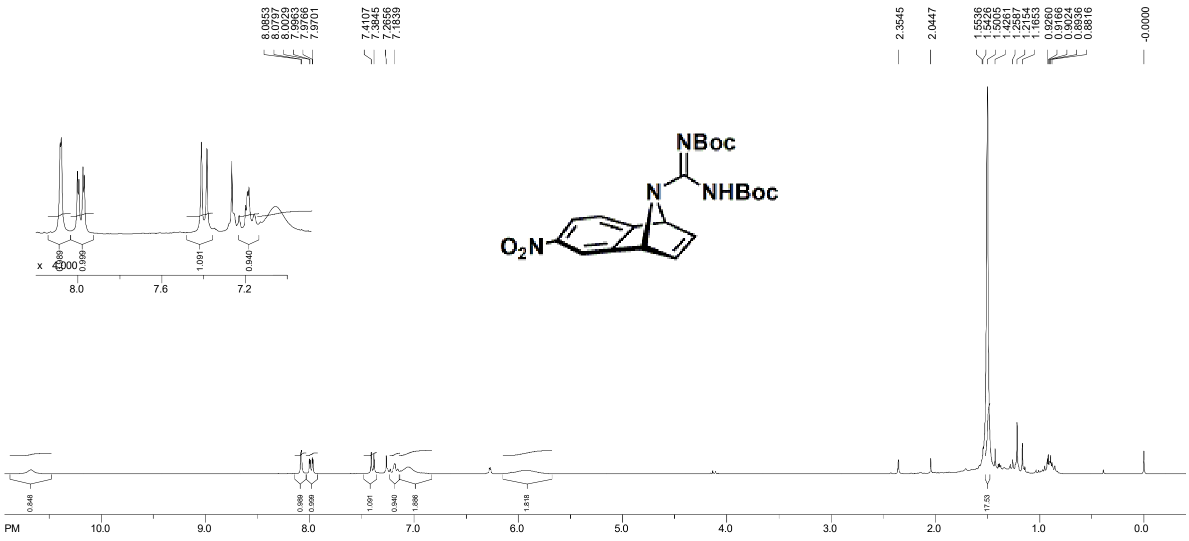

Figure S6. ^{1}H NMR (300 MHz, $CDCl_3$) spectrum of guanidine **23.**

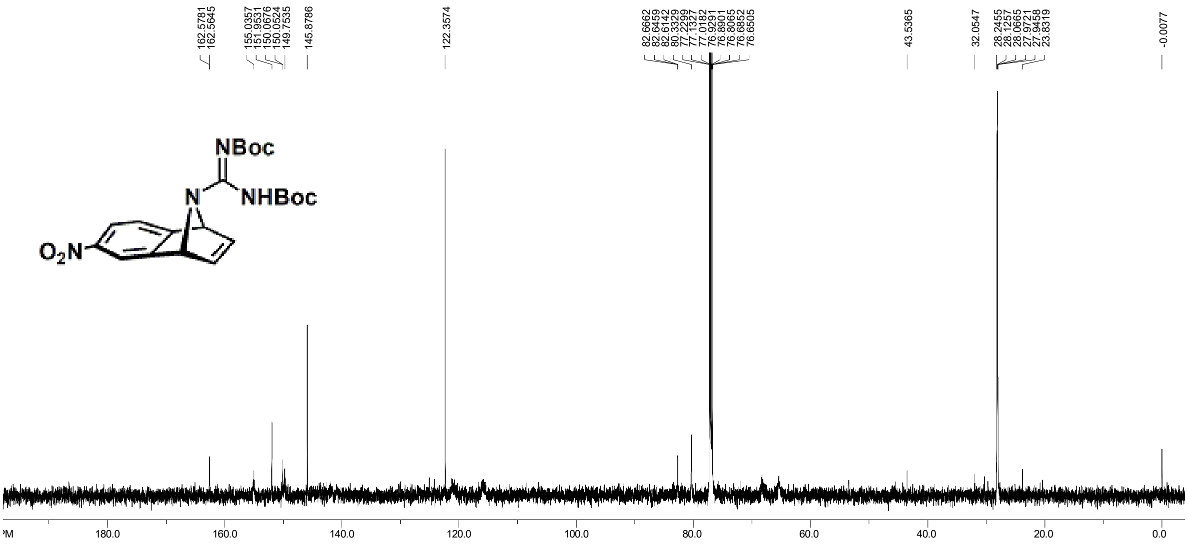

Figure S7. ^{13}C NMR (300 MHz, $CDCl_3$) spectrum of guanidine **23.**

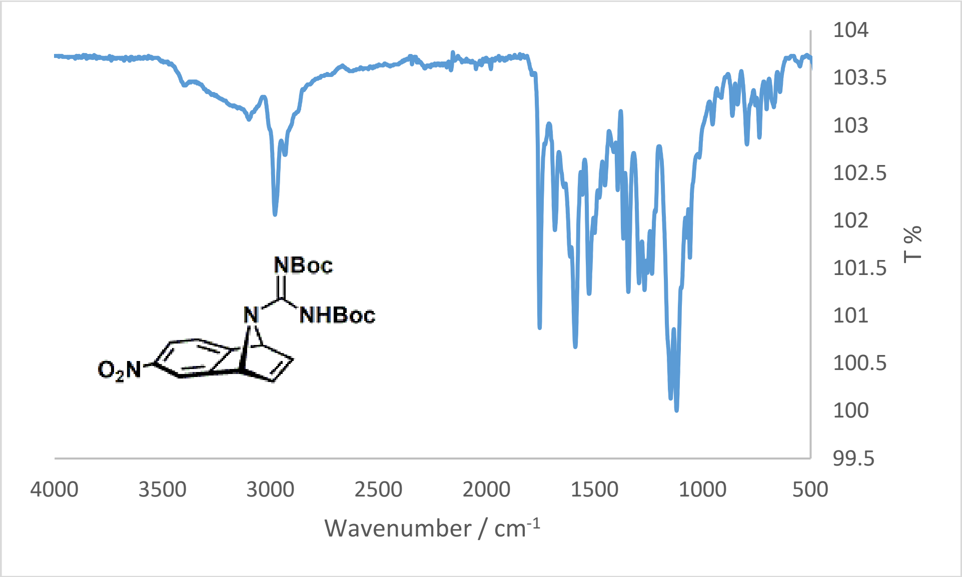

Figure S8. FTIR-ATR spectrum of guanidine **23.**

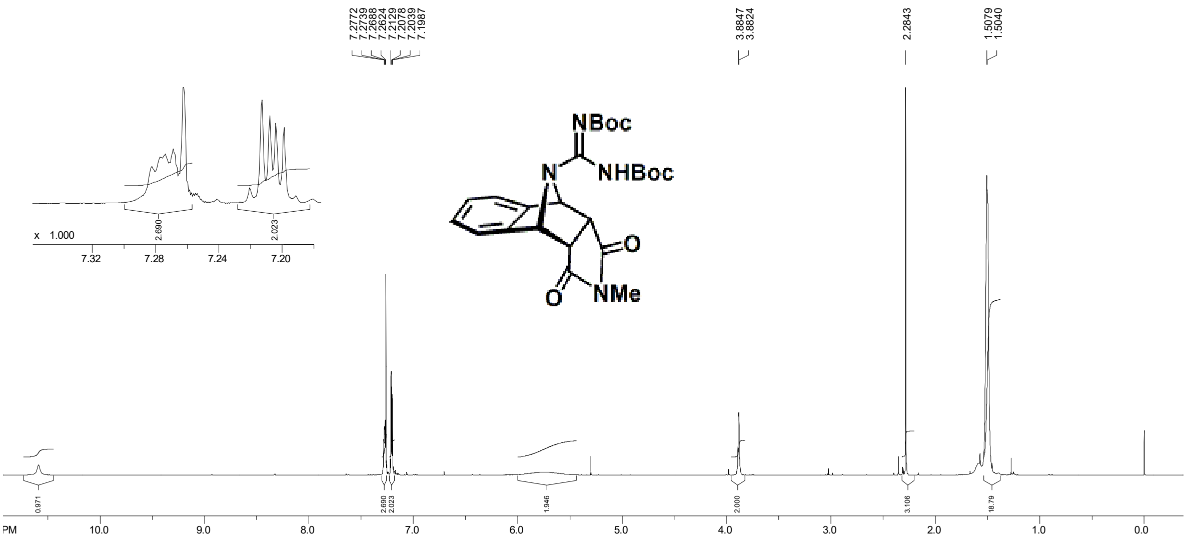

Figure S9. ^{1}H NMR (300 MHz, $CDCl_3$) spectrum of product **26.**

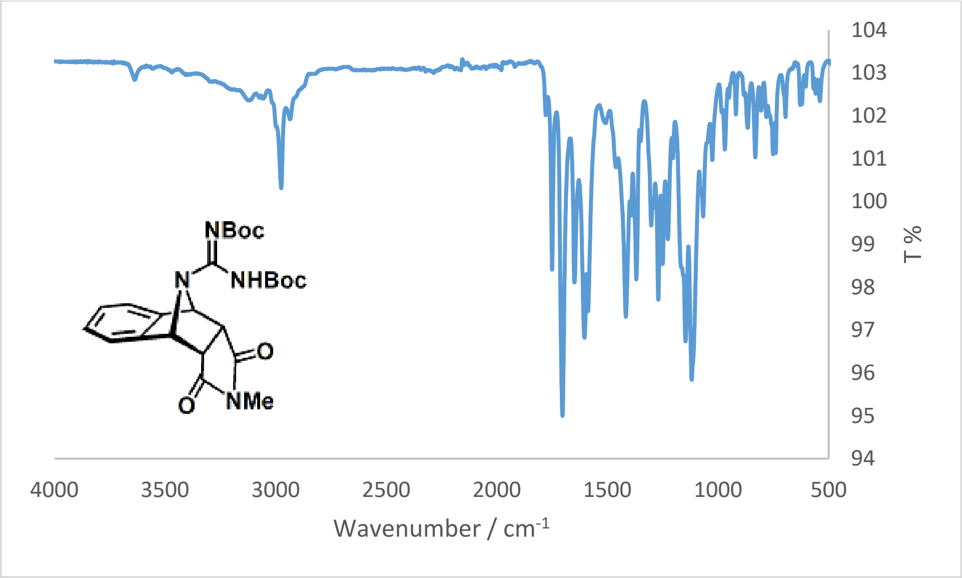

Figure S10. FTIR-ATR spectrum of product **26.**

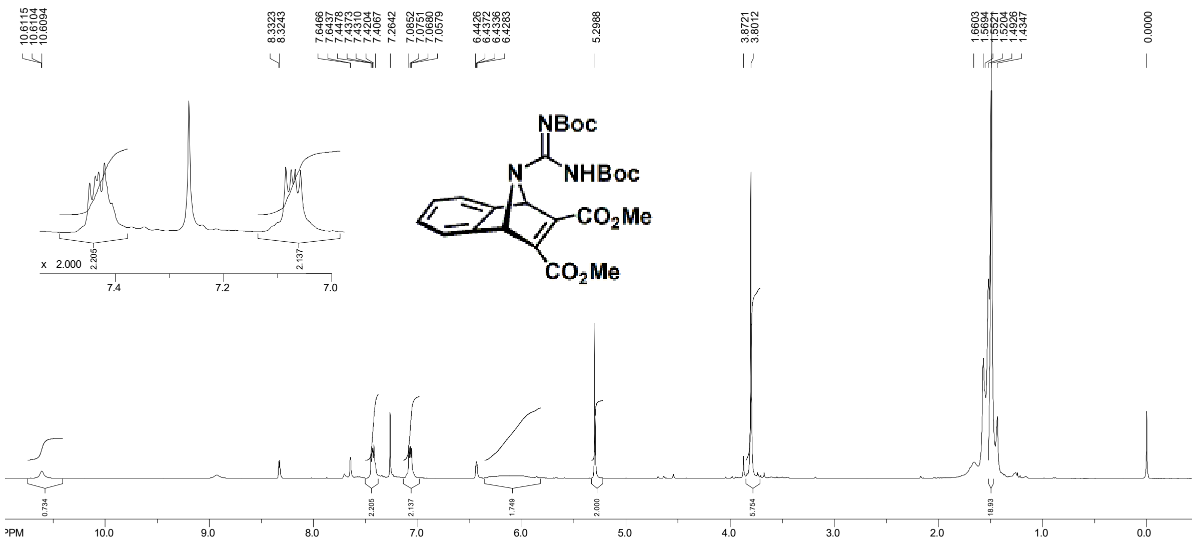

Figure S11. ^{1}H NMR (300 MHz, $CDCl_3$) spectrum of product **28.**

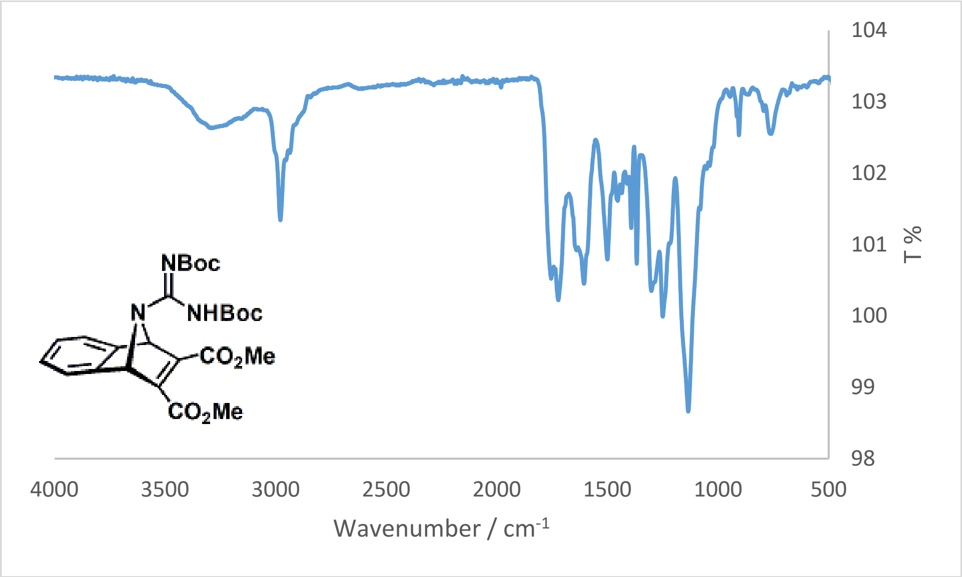

Figure S12. FTIR-ATR spectrum of product **28.**

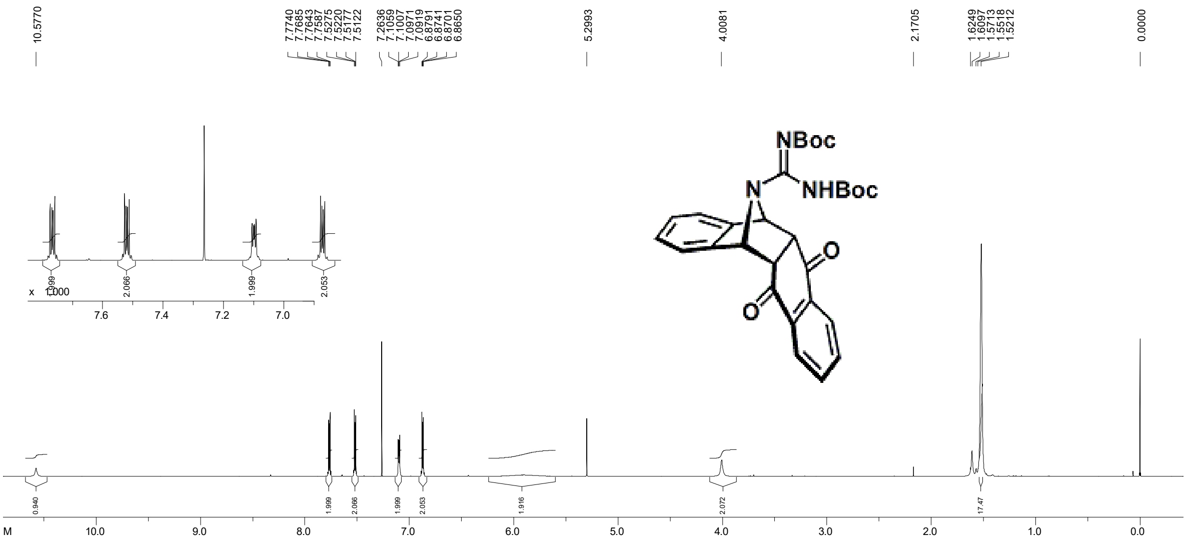

Figure S13. ^{1}H NMR (300 MHz, $CDCl_3$) spectrum of product **29.**

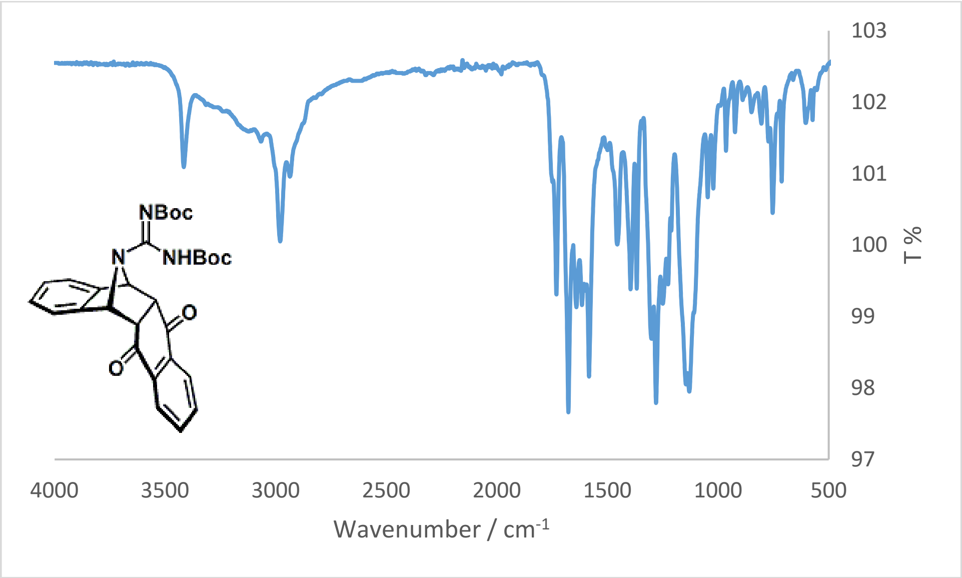

Figure S14. FTIR-ATR spectrum of product **29.**

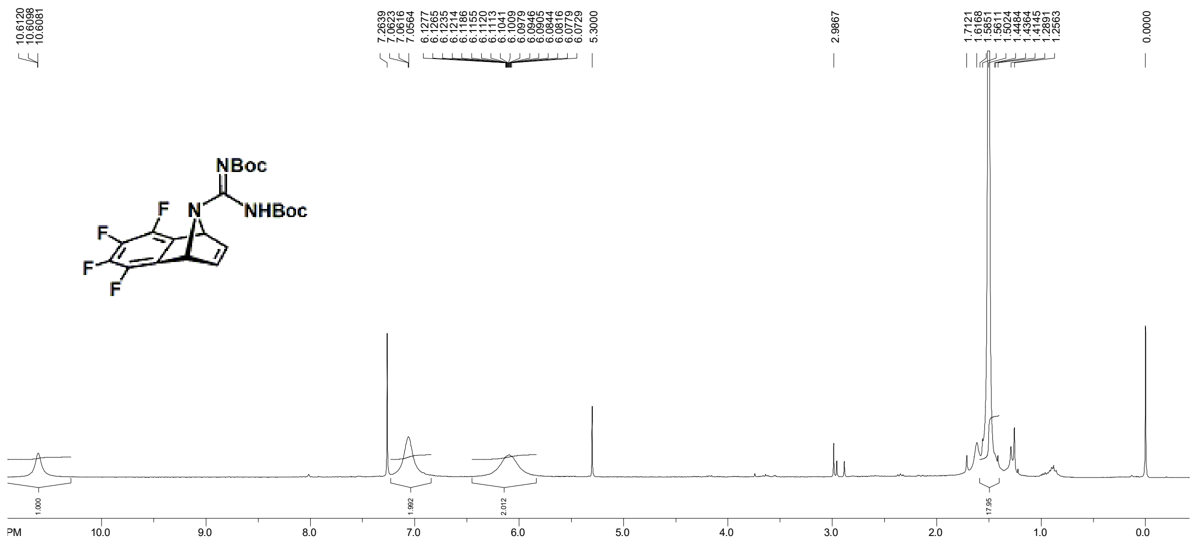

Figure S15. ^{1}H NMR (300 MHz, $CDCl_3$) spectrum of product **37.**

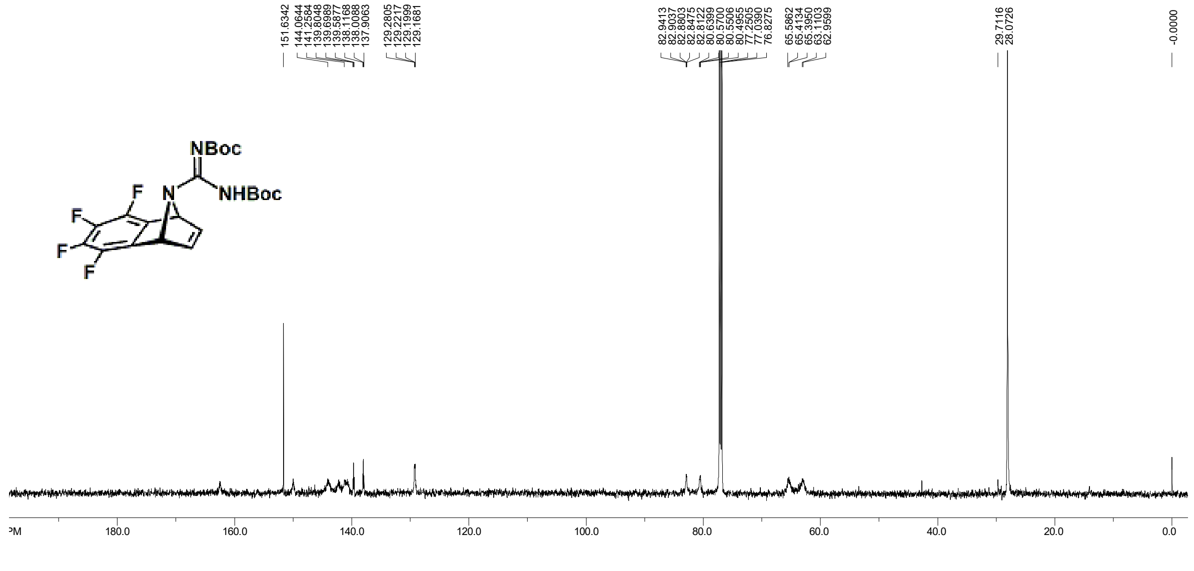

Figure S16. ^{13}C NMR (300 MHz, $CDCl_3$) spectrum of product **37.**

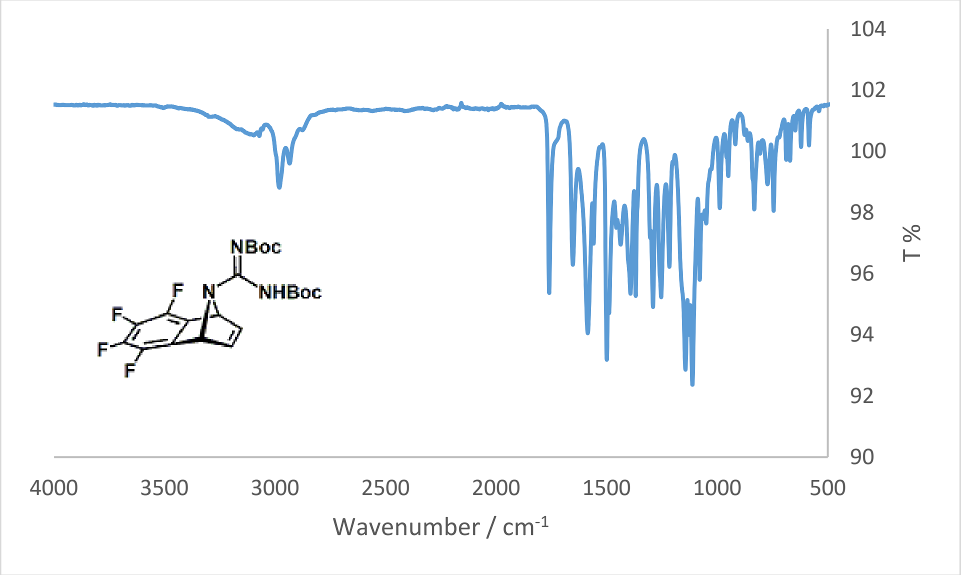

Figure S17. FTIR-ATR spectrum of product **37.**

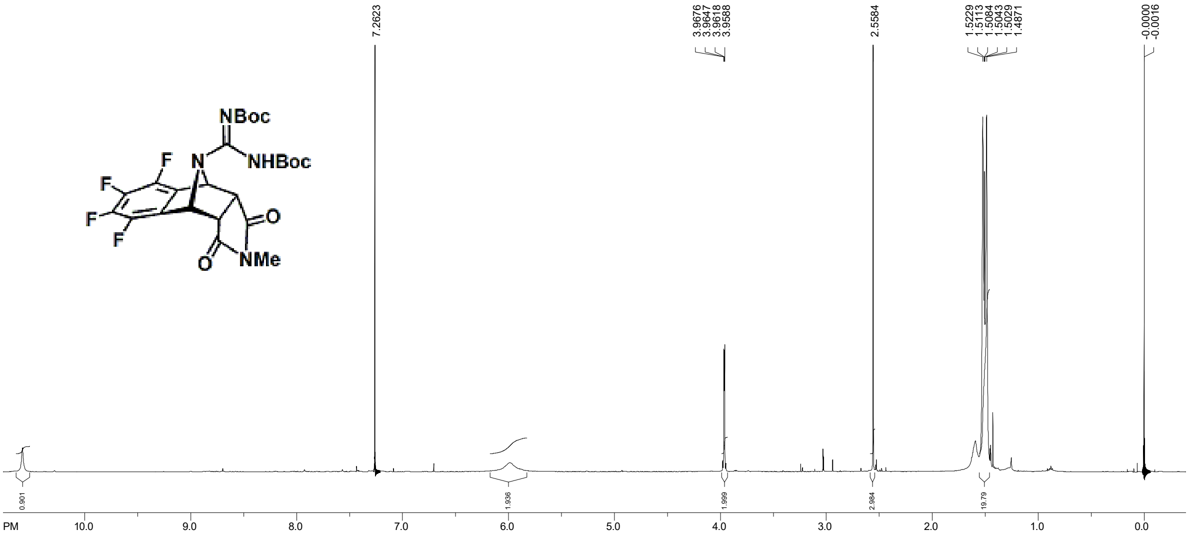

Figure S18. ^{1}H NMR (300 MHz, $CDCl_3$) spectrum of product **39.**

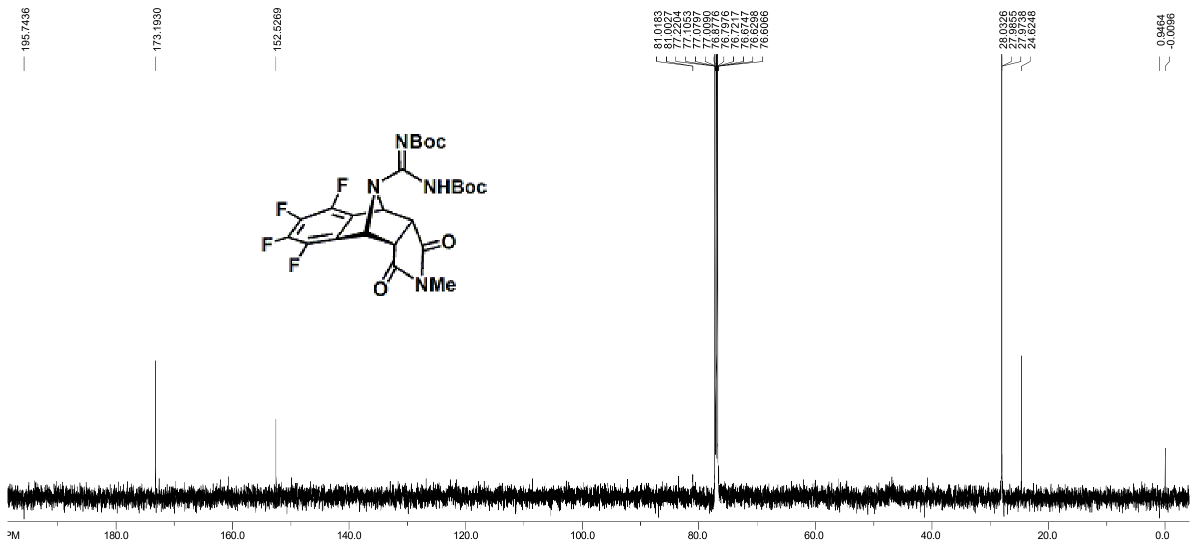

Figure S19. ^{13}C NMR (300 MHz, $CDCl_3$) spectrum of product **39.**

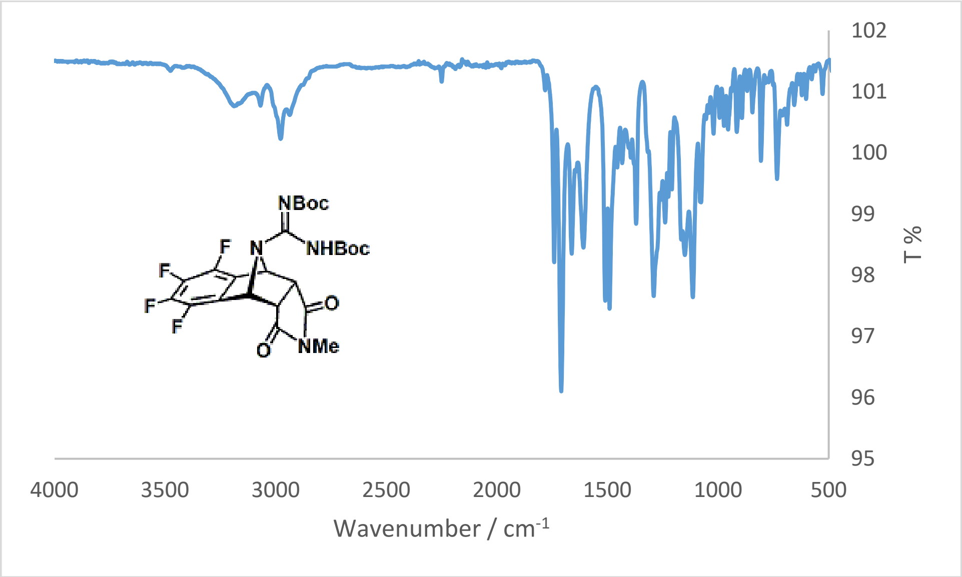

Figure S20. FTIR-ATR spectrum of product **39.**

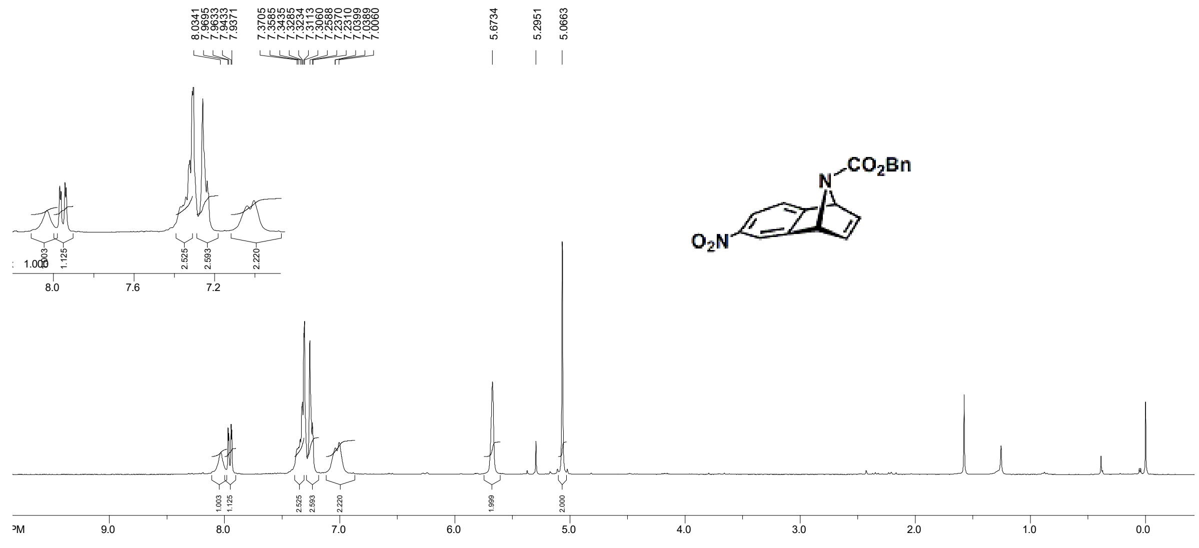

Figure S21. ^{1}H NMR (300 MHz, $CDCl_3$) spectrum of product **42.**

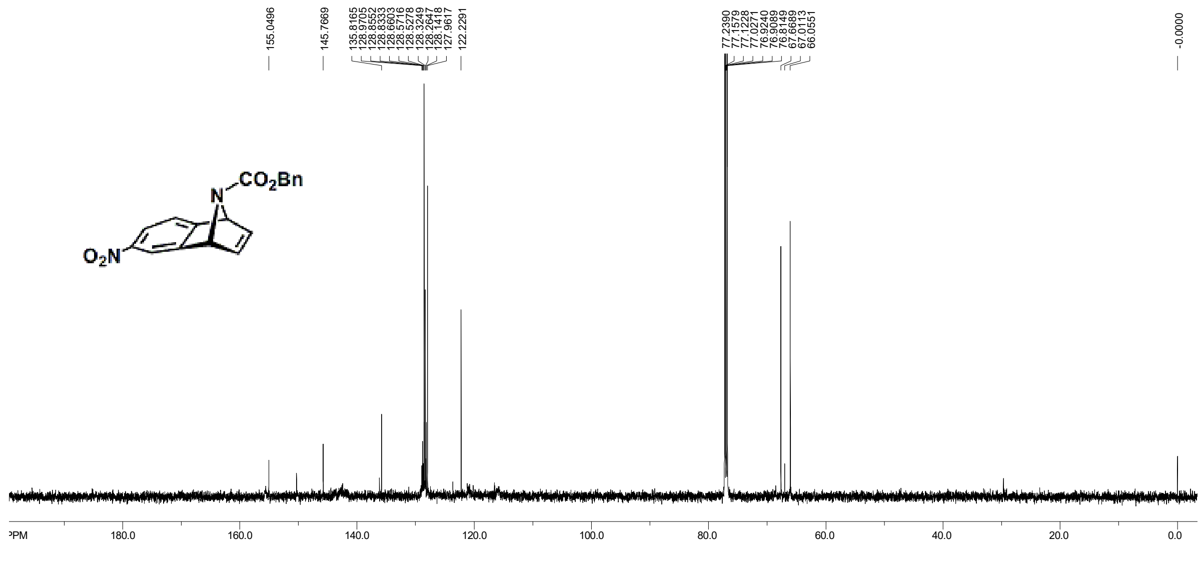

Figure S22. ^{13}C NMR (300 MHz, $CDCl_3$) spectrum of product **42.**

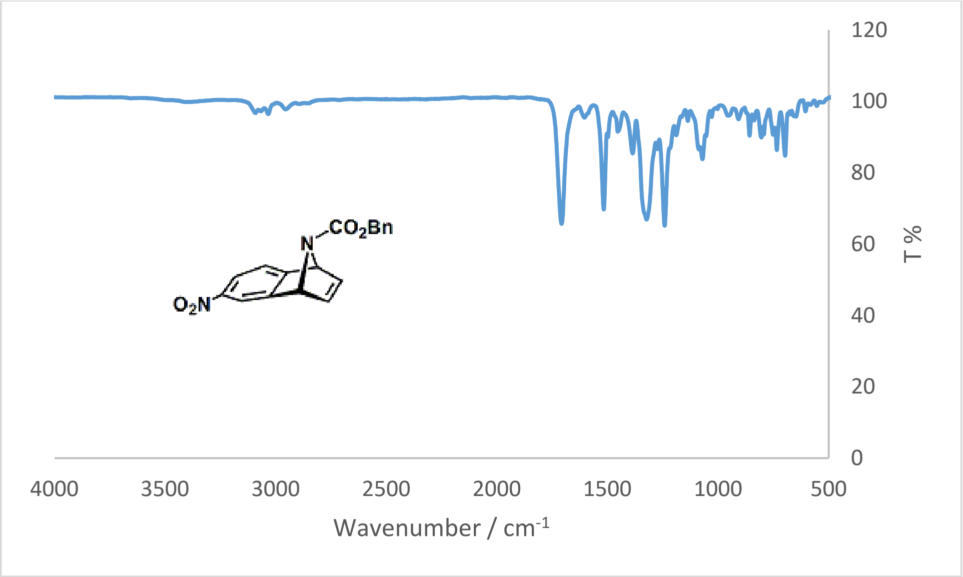

Figure S23. FTIR-ATR spectrum of product **42.**

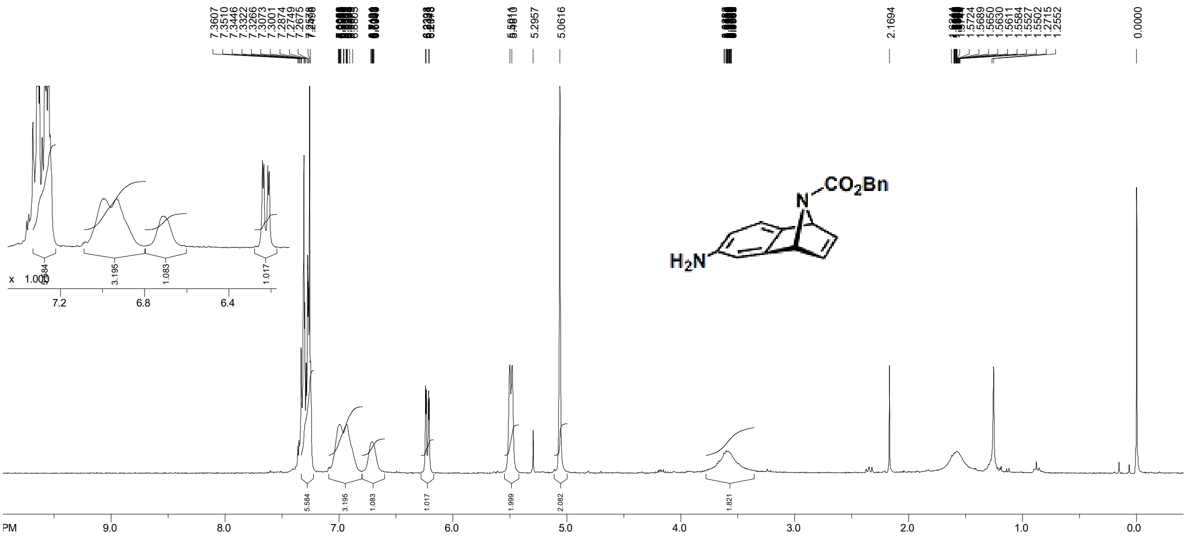

Figure S24. ^{1}H NMR (300 MHz, CDCl3) spectrum of product **43.**

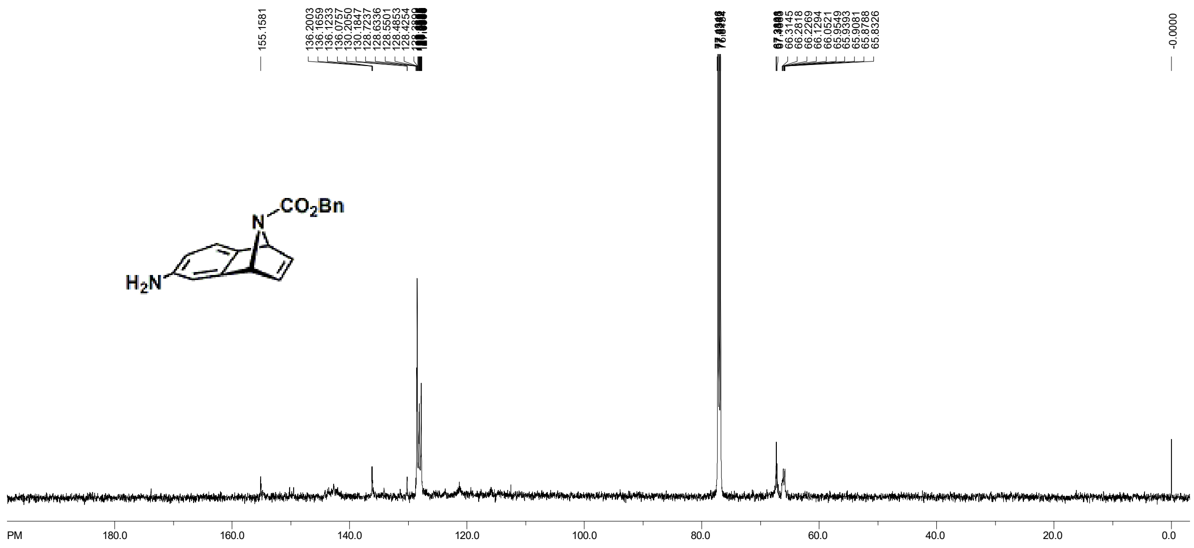

Figure S25. ^{13}C NMR (300 MHz, $CDCl_3$) spectrum of product **43.**

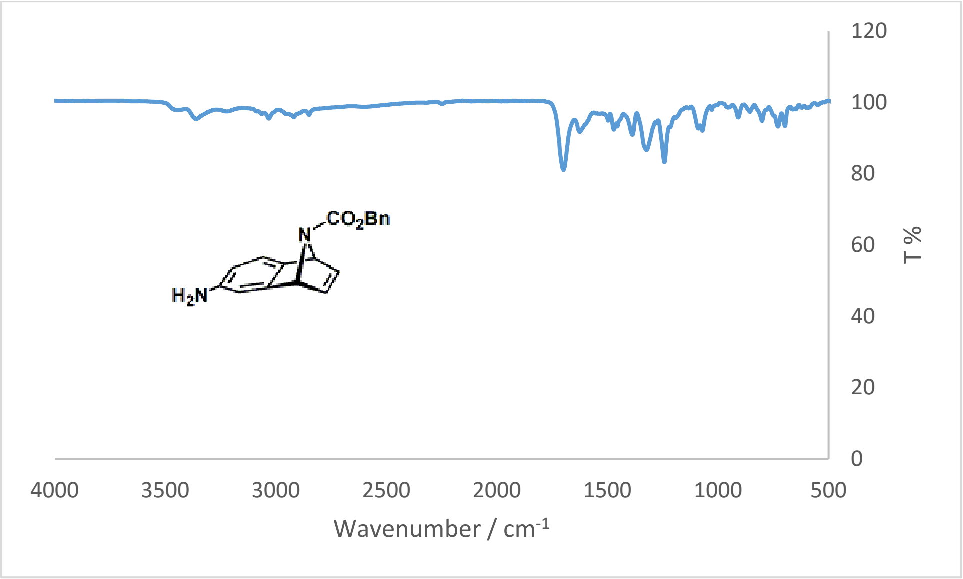

Figure S26. FTIR-ATR spectrum of product **43.**

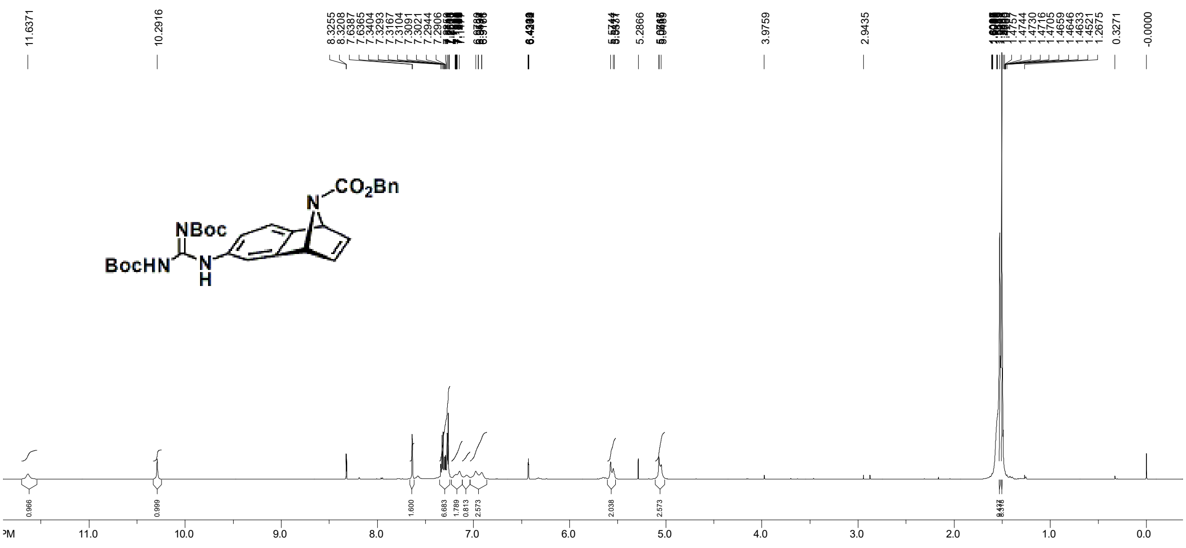

Figure S27. ^{1}H NMR (300 MHz, $CDCl_3$) spectrum of guanidine **44.**

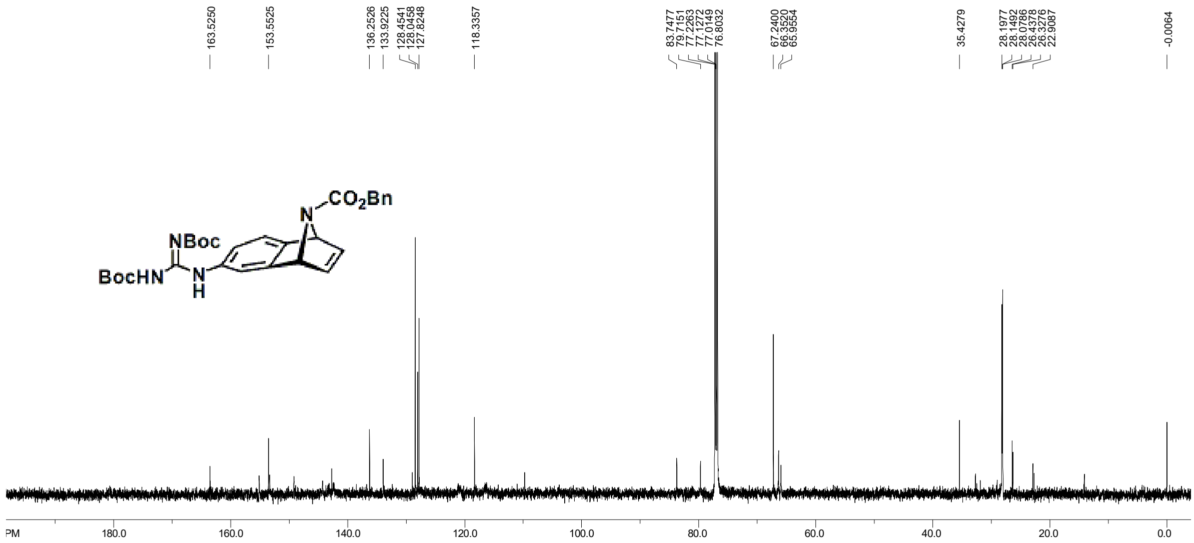

Figure S28. ^{13}C NMR (300 MHz, $CDCl_3$) spectrum of guanidine **44.**

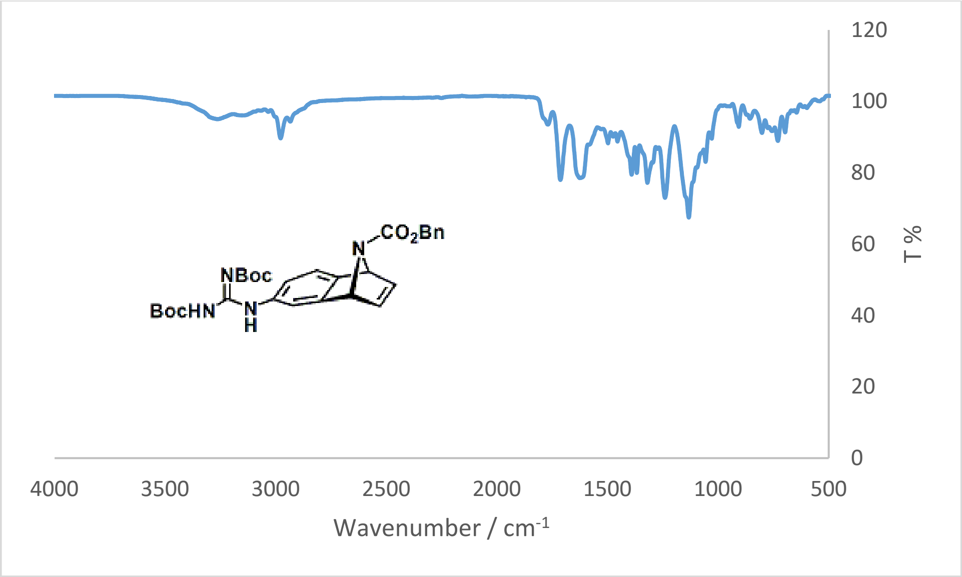

Figure S29. FTIR-ATR spectrum of guanidine **44.**

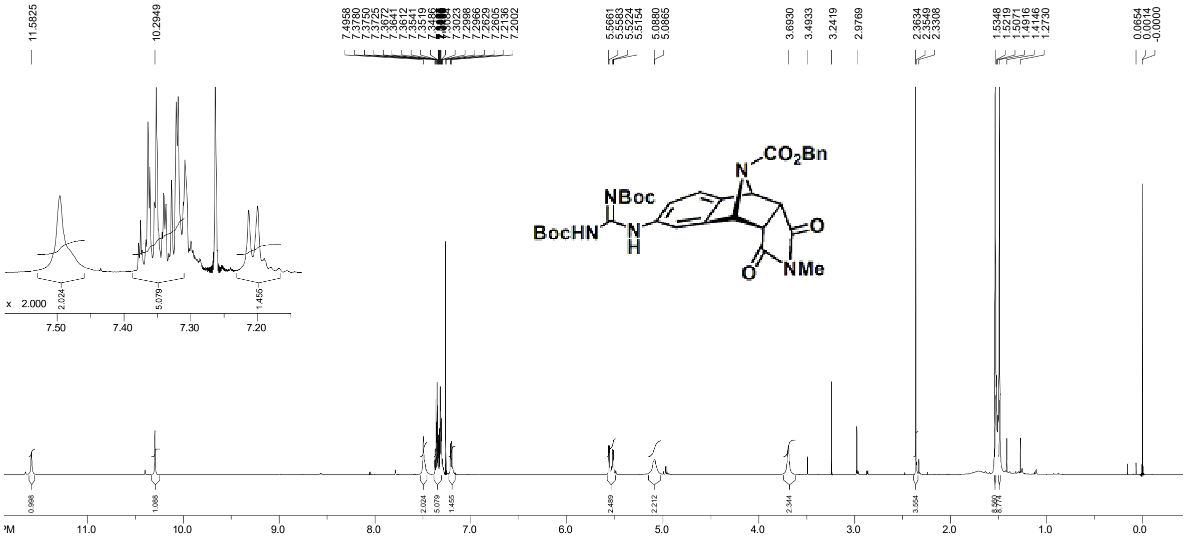

Figure S30. ¹H NMR (300 MHz, CDCl3) spectrum of product **46.**

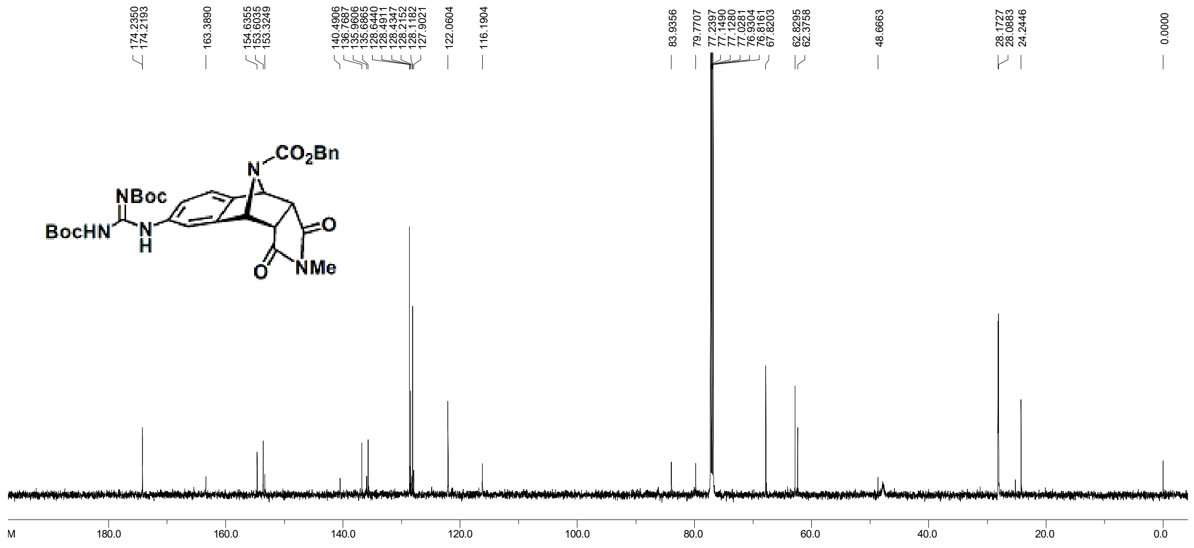

Figure S31. ^{13}C NMR (300 MHz, $CDCl_3$) spectrum of product **46.**

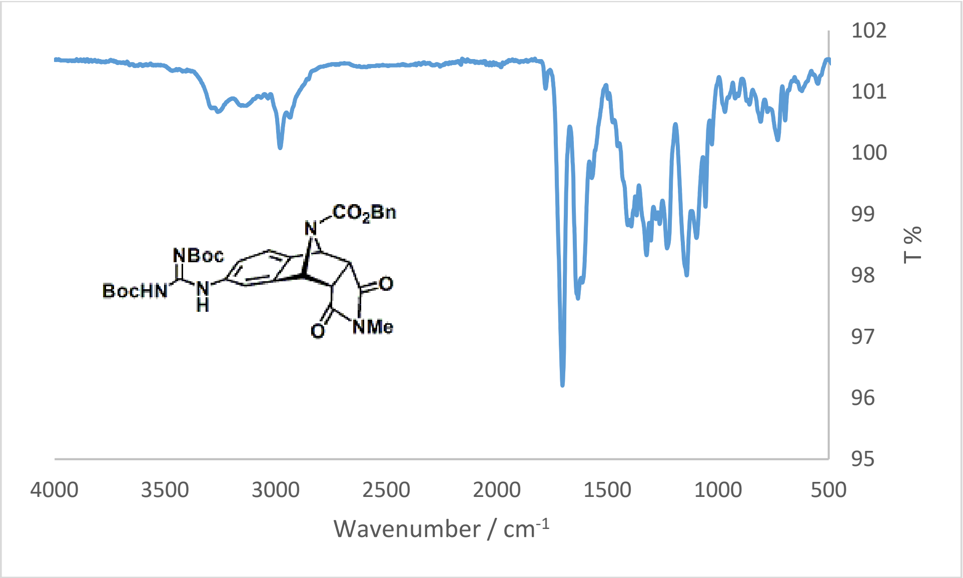

Figure S32. FTIR-ATR spectrum of product **46.**
